# Current Pharmacotherapy and Multi-Target Approaches for Alzheimer’s Disease

**DOI:** 10.3390/ph15121560

**Published:** 2022-12-14

**Authors:** Siew Lee Cheong, Jian Kai Tiew, Yi Hang Fong, How Wan Leong, Yew Mun Chan, Zhi Ling Chan, Ethan Wei Jie Kong

**Affiliations:** 1 Department of Pharmaceutical Chemistry, School of Pharmacy, International Medical University, No. 126, Jalan Jalil Perkasa 19, Bukit Jalil, Kuala Lumpur 57000, Malaysia; 2 School of Postgraduate Studies, International Medical University, No. 126, Jalan Jalil Perkasa 19, Bukit Jalil, Kuala Lumpur 57000, Malaysia

**Keywords:** Alzheimer’s disease, pathogenesis, pharmacotherapy, multi-target ligands, polypharmacology

## Abstract

Alzheimer’s disease (AD) is a neurodegenerative disorder characterized by decreased synaptic transmission and cerebral atrophy with appearance of amyloid plaques and neurofibrillary tangles. Cognitive, functional, and behavioral alterations are commonly associated with the disease. Different pathophysiological pathways of AD have been proposed, some of which interact and influence one another. Current treatment for AD mainly involves the use of therapeutic agents to alleviate the symptoms in AD patients. The conventional single-target treatment approaches do not often cause the desired effect in the disease due to its multifactorial origin. Thus, multi-target strategies have since been undertaken, which aim to simultaneously target multiple targets involved in the development of AD. In this review, we provide an overview of the pathogenesis of AD and the current drug therapies for the disease. Additionally, rationales of the multi-target approaches and examples of multi-target drugs with pharmacological actions against AD are also discussed.

## 1. Introduction

Alzheimer’s disease (AD) is a growing concern among communities nowadays. In the United States, there are more than five million Americans who are living with AD, with the majority of people 65 years old and older [1]. The Alzheimer’s Association Report estimated that the number of people affected by AD in the United States will be increased up to fourteen million by 2060 [1]. The disease, which is the most common cause of dementia, is a progressive and irreversible disorder of the brain that slowly deteriorates the brain function of an individual [2]. It progresses from preclinical, early- to moderate-stage, and finally late-stage disease. The early symptoms include mainly cognitive impairment, in particular memory loss. As cognitive function deteriorates, presentation of physical disabilities, such as the inability to walk, sit and eat indicates that the disease has progressed to the later stages [3]. Intracellular neurofibrillary tangles and extracellular amyloid β plaques are the hallmark characteristics found in the cortical and limbic areas of the brain that are associated with AD [4]. 

Generally, current treatment for the AD can be classified into two main categories based on the stages of the disease. For mild to moderate cases, galantamine, rivastigmine and donepezil as acetylcholinesterase inhibitors are indicated to provide temporary symptomatic relief among the patients [4]. Memantine, an N-methyl D-aspartate (NMDA) antagonist is used as a monotherapy to manage the symptoms in moderate to severe AD [4]. These drugs are mostly selective compounds that target individual proteins (“one compound–one target” approach), and are mostly aimed at restoring physiological acetylcholine levels. Nonetheless, various mechanisms of AD pathogenesis have been proposed to date, which are shown to overlap and influence one another [5]. This complexity challenges the dominant single-target approach in the treatment of AD. In fact, it has been widely recognized that the conventional single-target approach may not be adequately effective against AD that has a multifactorial origin involving a combination of genetic, metabolic, and environmental factors [5,6,7,8]. 

As a result, multi-target strategies have increasingly been considered as alternative options for the management of multifactorial AD in the past decades [9,10]. Amongst them, combination therapies based on a “cocktail drug–multiple targets” approach combining several drugs acting independently on different targets have been adopted to alleviate the symptoms of AD, such as a drug combination consisting of memantine and donepezil that has been used clinically to manage the symptoms in moderate to severe AD [4]. These drugs may act on the targets of different or the same pathways that are involved in the pathogenesis of AD [9]. Nevertheless, they are often associated with side effects due to drug–drug interactions, for instances bradycardia, atrioventricular block and psychosis [11] as well as varying pharmacokinetic profiles of each component drug [12].

Another multi-target strategy with a “one compound–multiple targets” approach has emerged and is regarded as a polypharmacological therapy for AD [9,10]. In such an approach, a single drug compound is designed to simultaneously target two or more specific proteins involved in the development of AD. The single ligand can beneficially eliminate side effects from interactions amongst drugs in the combination therapies with a more predictable pharmacokinetic profile compared to multiple drugs administered in combination [12]. Moreover, it also can enhance patient compliance with simple dosing schedules [13]. Hence, the multi-target drugs may represent a potential alternative to the therapeutic regimen of combination therapy in regulating disease progression. In the following sections, the pathogenesis of AD and the currently available drug therapies are discussed. In addition, the multi-target therapies based on polypharmacological ligands for the disease are elaborated. 

## 2. Pathogenesis of Alzheimer’s Disease

As AD is a complex and multifactorial disease, a clear understanding of the underlying pathogenesis of the disease is essential for developing effective therapeutic regimens. Most of the early onset, autosomal dominant AD cases are characterized by the presence of extracellular beta amyloid (Aβ) plaques in various regions of the AD patient’s brain, due to either overproduction or reduced clearance of Aβ peptides or both [14]. Studies have found that amyloid precursor protein (APP) is associated with the pathogenesis of AD. In the brain, a group of enzymes known as APP secretases, including α-secretases, β-secretases and γ-secretases work together to process the APP [14]. In the physiological pathway, the α-secretases process the APP and produce soluble APPα (sAPP-α), which can preclude the subsequent β-γ secretase activity [14,15]. Evidence has shown that the soluble APPα is neuroprotective; it allows proper synaptic signaling and maintains neuronal plasticity [16]. On the other hand, in the amyloidogenic pathway, the β-secretases, or BACE-1, yield soluble APPβ (sAPP-β) and a small carboxy (C)-terminal fragment (CTFβ); both are cut by the γ-secretases into insoluble and neurotoxic Aβ peptides [9]. The two dominant forms of Aβ peptides produced in AD are Aβ40 and Aβ42 with 40 and 42 amino acid residues, respectively [14]. These Aβ peptides tend to aggregate to form oligomers of Aβ (oAβ), which will further aggregate into insoluble amyloid plaques or senile plaques [5]. In the case of sporadic or late onset AD, expression of the apolipoprotein E4 (APOE_4_) gene has been found to be a factor that contributes to the pathogenesis. Studies have reported that individuals with expression of APOE_4_ have an increase in beta-amyloid deposition together with impaired memory [5].

Mitochondrial dysfunction is secondary to the primary pathologic event of AD, the production of Aβ [17]. The Aβ plaques are largely found in the mitochondria of neuronal cells in AD patients; it can modify the structure of mitochondria and block the ion channels, thus interrupting calcium homeostasis [5,16]. This results in decreased mitochondrial respiration and ATP synthesis [5]. Events such as elevated mitochondrial fission and diminished mitochondrial fusion are also observed following exposure to Aβ plaques [5]. Such senile plaques are also found to cause an increase in oxidative stress due to intracellular formation of reactive oxygen species (ROS) from the mitochondria, which leads to a low energy metabolism rate, and eventually neuronal cell apoptosis with release of cytochrome c [5,16]. 

As opposed to the Aβ plaques, neurofibrillary tangles (NFTs) are another hallmark of the disease that is detected intracellularly in the brain of AD patients. This phenomenon can be illustrated by the Tau hypothesis. Tau (τ), a microtubule-associated protein, has a role in stabilizing the microtubules [16]. However, the Aβ42 that accumulates to high levels in the brain, increases the risk of hyperphosphorylation of the tau protein [18], which is regulated by several kinases, such as glycogen synthase kinase 3 (GSK-3β) and cyclin-dependent kinase 5 (CDK5) [16,19]. As such, the structure of the microtubule is disrupted and becomes unstable as the subunits are dissociated from itself without the support of the tau protein. The phosphorylated tau proteins clump together and form straight, insoluble, and fibrillary tau filaments; they are then aggregated into deposits called NFTs in the cytoplasm, which are neurotoxic. This leads to synaptic loss and affects the signaling process between neurons. As a result, apoptosis of the neuronal cells ensues [16]. 

The formation of both neurotoxic Aβ plaques and NFTs can increase oxidative stress and provoke synaptic damage. This attracts microglia to the vicinity of the plaques which act as resident phagocytes for clearance of both Aβ and the NFTs. The activated microglia following the binding of Aβ and the NFTs to its cell-surface receptors induce production of pro-inflammatory cytokines and other mediators for phagocytosis [20,21]. However, elevated cytokine levels under chronic inflammation leads to downregulation of the phagocytic receptor expression on microglia, resulting in an ineffective Aβ clearance [16]. The Aβ-induced microglia are also found to generate ROS that can cause further oxidative damage to the neuronal cells. Consequently, this has led to a continuous cycle of microglia-mediated neuroinflammation and neuronal cell death [22]. 

The pathogenesis of AD is also suggested to be linked to the NMDA receptor. Essentially, binding of the glutamate excitatory neurotransmitter to the NMDA receptors allows regulation of synaptic plasticity and provides normal learning and memory functions through a process called long-term potentiation (LTP) [23]. In AD, an excitotoxicity hypothesis is proposed, in which the NMDA receptors are overactivated by the Aβ plaques; and this contributes to excessive Ca^2+^ fluxes, leading to excitotoxicity and impairment of the mitochondrial energy metabolism. Thus, free ROS production is favored, subsequently causing an increase in oxidative stress and altered synapse function [24]. Apart from this, Kochahan and co-workers also reported that Aβ oligomers induced spine loss and have resulted in the reduction of the amount of glutamate receptors available for binding, hence inhibiting the LTP at the hippocampus and other regions of the brain. Without proper excitatory transmission through NMDA receptors and normal synaptic function, it promotes further progression of AD. Therefore, the NMDA receptors have been regarded as potential targets of interest for tackling the cognitive impairment occurring in AD [23]. 

Additionally, a cholinergic hypothesis has been postulated for the pathogenesis of end-stage AD [25,26]. Based on the hypothesis, AD may be due to the loss of central cholinergic neurons that leads to deficiency of a neurotransmitter responsible for memory and learning, known as acetylcholine (ACh). Research also showed that the AD brain has notably diminished activity of choline acetyltransferase (ChAT) involved in acetylcholine synthesis and reduced metabolism of the acetylcholinesterase (AChE) [25]. Nonetheless, cholinergic depletion is not the only factor that causes the decline in cognitive functions [26]. Instead, aging is also a factor that causes natural loss of ACh and impairs the ability of cholinergic neurons to release ACh for neurotransmission; this increases the susceptibility of hippocampus to damages from other central nervous system complications, such as stress, seizure, or stroke. Ultimately, this has brought about the memory and cognitive deficits in AD [26,27]. Aside from the cholinergic pathways, AChE is also found to be associated with the non-cholinergic function via AChE-induced Aβ aggregation that can eventually lead to neurotoxicity [28]. 

**Figure 1:**
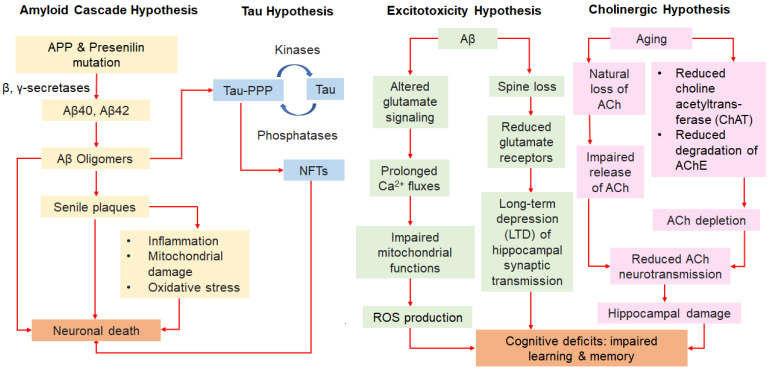
Flowchart of different hypotheses on pathogenesis of Alzheimer’s disease.

In general, multiple hypotheses have been associated with the pathogenesis of AD (Figure 1). Researchers have put in much effort to investigate the mechanisms involved in the AD pathogenesis, which have helped to accelerate the discovery of potential therapeutic agents for the management of AD. 

## 3. Current Drug Therapies for Alzheimer’s Disease

Research conducted on AD thus far has improved the knowledge on the pathophysiology of the disease. Nevertheless, there are only a few medications approved by the Food and Drug Administration (FDA) to manage the disease. These medications are mainly used to improve the symptoms of AD, such as cognitive and global functioning; they are unable to delay the progression or treat the underlying causes of AD [29,30,31]. Currently, there are five main pharmacotherapies for AD based on two drug classes, namely AChE inhibitors (rivastigmine, donepezil, galantamine) and NMDA receptor antagonists (memantine), as well as a combination therapy of an acetylcholinesterase inhibitor with memantine. 

**Figure 2:**
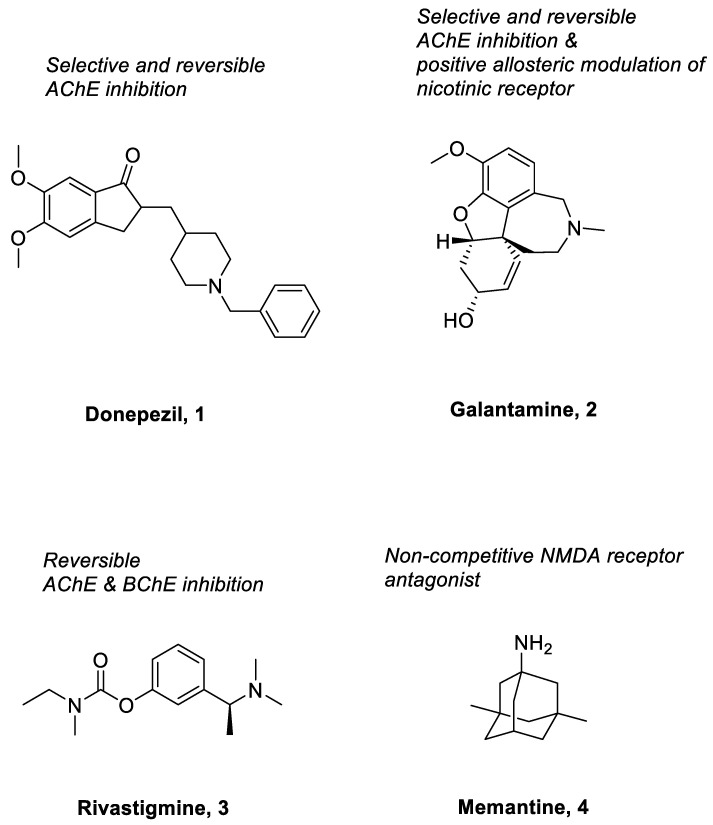
Current pharmacotherapy for Alzheimer’s disease.

Donepezil (**1**) (Figure 2), considered as the first line treatment for AD, is a second generation of AChE inhibitor along with rivastigmine and galantamine. It is a highly selective, reversible and non-competitive AChE inhibitor, which is slowly absorbed from the gastrointestinal tract and has a relative long half-life (50 to 70 h) [32,33]. Basically, it acts by increasing the concentration of acetylcholine in the synaptic cleft of the hippocampus through inhibition of AChE and causes stimulation of brainstem reticular formation that leads to an increase in hippocampal theta rhythm amplitude [33]. In China, donepezil was approved for use in mild to moderate AD in 2006 and severe AD in 2017 [33]. A study that involved 603 patients, by Black et al., concluded that there was significant improvement on the Alzheimer’s Disease Assessment Scale-Cognitive Subscale (ADAS-cog) scores at all time-points [34]. It was further supported by another 12-week, randomized, multinational study done by Wilkinson et al., which involved 111 patients with mild to moderate AD. The study consistently showed that there was comparable improvement on the ADAS-cog scores. Generally, the use of donepezil was well tolerated in patients with mild to moderate AD due to lesser treatment-emergent adverse effects when compared with the use of rivastigmine [35]. The recommended dose of donepezil for mild and moderate AD is 5 mg once daily and it may be increased up to 10 mg/day after four to six weeks. In contrast, the recommended dose of donepezil for moderate to severe AD is 10 mg or 23 mg once daily. A randomized, controlled trial comparing the benefits of treating moderate to severe AD using 10 mg and 23 mg daily doses of donepezil concluded that a 23 mg daily dosing of donepezil showed better cognitive benefits in treating moderate to severe AD [36]. Common adverse effects, such as nausea, diarrhea, agitation and dizziness associated with donepezil are generally of mild to moderate intensity and can be resolved without the need for discontinuation of medication [34]. 

Galantamine (**2**) (Figure 2), a medication with dual mechanisms of action, is a rapidly reversible acetylcholinesterase inhibitor and a positive allosteric modulator of nicotinic receptors [37]. A study by Wallin et al. was previously conducted to evaluate the long-term effect of galantamine treatment in 280 AD patients [38]. The study showed marked improvement in terms of cognitive assessment (based on the Mini-Mental State Exam (MMSE) and ADAS-cog scores, with a mean change from baseline of 2.6 points and 5.6 points, respectively, upon three years of galantamine treatment). This was significantly better than the predicted annual decline in scores for untreated patients in both parameters (2 to 4 points in MMSE score and 6.7 points in ADAS-cog score) [38]. It is supported by another study by Thavichachart et al., in which two-thirds of patients (67.8%) reported improvement in ADAS-cog score, especially in those with mild and moderate severity of AD [37]. Slow dose escalation was well tolerated and fewer adverse effects were reported when patients received 16 mg/day of galantamine versus 24 mg/day of galantamine [37]. The recommended dose of galantamine for mild AD is 16 mg/day, while 24 mg/day is more beneficial for the patients with moderate AD [39]. However, several adverse effects are encountered by patients during the galantamine treatment, including nausea (12%), weight loss (11%), dizziness (7%) and vomiting (5%). Fortunately, those were mild- to moderate- intensity adverse events that showed no clinical changes from baseline in all aspects [37]. 

Rivastigmine (**3**) (Figure 2) is another acetylcholinesterase inhibitor that is used for the management of AD. Unlike donepezil and galantamine which selectively inhibit the AChE, rivastigmine acts by inhibiting both the AChE and butyrylcholinesterase (BuChE) in the brain [39,40]. It has low protein binding, and hence shows minimal potential interaction with other drugs; this makes it a more suitable medication for those elderly who take many different medications concurrently [40,41]. A study conducted by Rösler et al. concluded that a high dose of rivastigmine (6–12 mg/day) showed marked improvement in terms of ADAS-cog, global function and progressive deterioration scale (PDS) scores in the AD patients when compared to the placebo group. More patients (24%) in the higher dose group had improved by 4 points or more in ADAS-cog score than those patients in the placebo group (16%). The same was observed with the global function (37% versus 20%) and PDS (29% versus 19%) whereby more patients in the higher dose group had significant improvement than those in the placebo group [42]. Another study by Karaman et al. stated that long term rivastigmine treatment was well tolerated and improved the cognitive and functional symptoms (such as non-epileptic attacks and weakness) in AD patients [43]. Based on the ADAS-cog score, only 18.3% of AD patients encountered a reduction of 4 or more points when they were on rivastigmine treatment, in contrast to 45% of placebo-treated patients who experienced a reduction of at least 4 points on the ADAS-cog scale [43]. The MMSE also showed that the AD patients treated with rivastigmine had a better score than those receiving placebo. There was improvement of 0.20 points from baseline in those patients treated with rivastigmine compared to those receiving placebo with deterioration of 1.2 points from baseline [43]. The recommended dose of rivastigmine in mild to moderate AD patients ranges from 6 to 12 mg/day orally in two separate doses. The patients should start at 1.5 mg BD and increase the dose in 1.5 mg increments as tolerated [40]. Generally, the adverse effects are not severe and can be resolved upon slower dose escalation. The most common adverse effects reported are nausea (16.6%), vomiting (12.5%), dizziness, anorexia and headache (8.3%) in those AD patients treated with rivastigmine [43]. 

Besides the AChE inhibitors, memantine (**4**) (Figure 2) is another medicine approved to manage the symptoms of AD. It is a voltage-dependent and non-competitive NMDA receptor antagonist, which selectively binds to NMDA receptor-operated calcium channels [44,45,46]. Under normal conditions, activation of the synaptic NMDA receptors induces plasticity and enhances the survival of neuronal cells [47,48,49]. However, excessive activity of the NMDA receptor is detrimental as it can cause excitotoxicity of the neurons [48,49,50]. When this occurs, the neuronal cells will undergo apoptosis, leading to neuronal dysfunction. Memantine inhibits the effects of overactivated NMDA receptors, thus reducing the apoptosis of neuronal cells and preventing neuronal damage. It has been shown to improve the cognitive functions of AD patients in all stages. A study conducted in 2011 by Schulz et al. concluded that a 20 mg once daily regimen of memantine significantly improved the cognition and functional communication in AD patients [51]. It is usually prescribed in patients with moderate to severe AD, or in mild to moderate AD patients who cannot tolerate acetylcholinesterase inhibitors [52]. The initial dose of memantine is 5 mg daily, followed by steady weekly increments of 5 mg, and a maximum dose of 20 mg daily. It is better tolerated than the acetylcholinesterase inhibitors, although cases of dizziness, headache, somnolence, constipation, and hypertension have been reported as side effects [52].

On top of the monotherapy with either AChE inhibitors or NMDA receptor antagonists, a combination therapy consisting of an AChE inhibitor and memantine is another treatment option for AD. In 2014, a fixed dose combination (FDC) of donepezil and memantine was approved as a pharmacological management option for moderate to severe AD [53]. Concurrent administration of an AChE inhibitor with memantine is believed to have synergistic effects in alleviating the symptoms of AD due to the complementary mechanism of actions. This combination has been shown to be effective in patients with an advanced stage of AD. A study by Tariot et al. suggested that addition of memantine in moderate to severe AD patients receiving stable doses of donepezil is beneficial [54]. The results favored the group of subjects receiving both donepezil and memantine at the same time. Atri et al. also analyzed the cumulative additive benefits of the memantine–donepezil combination over component monotherapies in moderate to severe Alzheimer’s dementia using area-under-curve (AUC) analysis [55]. The study found that the AUC of subjects receiving the donepezil–memantine combination had significant improvement compared to those receiving the monotherapy, thus indicating the additive effect of the combination therapy. Currently, such a drug combination is available in fixed doses of donepezil–memantine capsules. The recommended dose of donepezil–memantine FDC capsules is 28 mg memantine and 10 mg donepezil daily for both the moderate and severe AD patients [55]. A study has demonstrated that these capsules are bioequivalent to the concurrent administration of each individual component [56]. Furthermore, a fixed-dose capsule of donepezil and memantine may also enhance the adherence of patients to the treatment regimen. 

## 4. Multi-Target Approaches in the Discovery of Polypharmacological Ligands for Alzheimer’s Disease 

Most of the therapeutic drugs available on the market are single-target drugs indicated for various diseases. However, these single-target drugs have increasingly been found to be ineffective against diseases with a multi-factorial pathogenesis, such as Alzheimer’s disease. Indeed, the single-target FDA-approved drugs as illustrated in the previous section are the common therapeutic options for AD; unfortunately, these drugs are only effective in alleviating the symptoms of AD, but not in halting the disease progression. It has been suggested that drugs targeting multiple pathological pathways or targets might be another option to manage the disease progression of AD. Although combination therapy comprising two single-target drugs has been used currently to treat AD, such a therapy may lead to an increased incidence of adverse effects and a risk of drug resistance [11]. Combination of several drug molecules may also give rise to different degrees of bioavailability and pharmacokinetic profiles from each drug component [12,57]. 

As such, the focus has gradually shifted towards the design of a single ligand that modulates two or more specific targets of interest simultaneously, namely a polypharmacological ligand. In this case, the chances of encountering undesirable side effects are less when one ligand is used, as compared to using two or more ligands. In addition, a ligand that targets only one protein is more susceptible to resistance due to mutations in the active site of the target, thus substantially reducing binding affinity and efficacy of the ligand. Conversely, resistance to a compound targeting multiple proteins would require the unlikely occurrence of concurrent mutations that appear in the multiple protein targets [58]. Risk of drug–drug interactions is also lower in comparison to that of the combination therapy as only one compound is present; furthermore, patient medication compliance will be improved due to simplification of dosage regimen [59]. 

As AD progresses, it requires different treatment approaches to intervene in the underlying sub-pathologies. There are three main stages of intervention depending on the progression of AD, namely primary prevention, secondary prevention, and symptomatic treatment [59,60,61]. Primary prevention includes interventions targeting risk factors such as hypertension, diabetes, and dyslipidemia, which can lead to pathophysiological changes, for example Aβ plaque formation [62]. In secondary prevention, therapeutic interventions involve drugs targeting Aβ and Tau pathology, or neuroinflammation; AD patients at this stage present with the main hallmarks of AD, such as Aβ and tau aggregates or neuroinflammation, even though cognitive function is retained [6,62,63,64,65]. Lastly, the symptomatic treatment targets patients with impaired cognition, and compounds target impaired neurotransmission, for instance, AChE inhibitors and NMDA antagonists are of relevance [66]. It is therefore important to aim at the AD sub-pathologies that occur contemporaneously [59], especially for achieving simultaneous targeting by a polypharmacological ligand.

**Figure 3:**
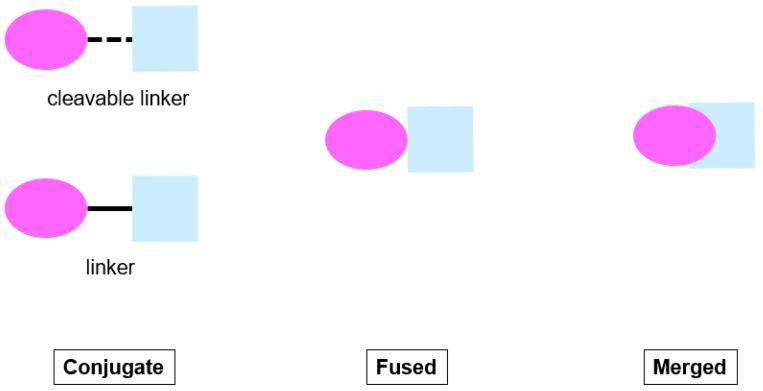
Drug design strategies of polypharmacological ligands.

There are basically three different strategies for generating polypharmacological ligands, namely knowledge-based/medicinal chemistry-based, biological screening-based and virtual screening-based approaches. Most of the polypharmacological ligands are derived upon knowledge-based/medicinal chemistry-based approaches that rely on the biological data of existing drugs from literature or commercial sources. There are three types of polypharmacological ligands under this approach in general, which are classified as conjugate, fused, and merged ligands [67] (Figure 3). The conjugates are designed and synthesized to be composed of pharmacophoric structures which are connected by a metabolically stable linker or a cleavable linker to be metabolized with release of individual active structures in vivo that interact independently with each target. In the fused ligands, the pharmacophoric structures are essentially joined at the junctions without the use of a linker. The pharmacophores of the structures do not overlap; they are combined via direct reactivity of functional groups of the pharmacophores. For the merged ligands, they have the maximal overlap of pharmacophoric features from the individual active components, which ultimately give rise to smaller and simpler molecules. Among the three types of ligands, the conjugates have the highest molecular weight, followed by the fused ligands and then the merged ligands [67]. 

A recent study by González and co-workers has illustrated a list of compounds, of which the chemical moieties can be used as scaffolds for the development of polypharmacological lead compounds for AD [68]. Each of the compounds possesses one or more biological activities that are relevant to AD, thus conferring different therapeutic benefits. Examples of compounds, their corresponding biological activities and chemical moieties [68] are summarized in Table 1. 

Over the past decades, research on the pathogenesis of AD has revealed various potential therapeutic targets, including AChE, BuChE, BACE-1, highly conserved threonine-serine kinase GSK-3β, MAO, metal ions in the brain, NMDA receptor, 5-HT receptor, serotonin transporter (SERT), cannabinoid receptor subtype 2 (CB_2_R), histamine H_3_ receptor, phosphodiesterases (PDEs), advanced glycation end products (AGEs), fatty acid amide hydrolase (FAHH), nuclear factor erythroid 2-related factor 2 (Nrf2), cyclooxygenase-2 (COX-2), 5-lipoxygenase (5-LOX) and SH2 domain containing inositol 50-phosphatase 2 (SHIP2). Each of the therapeutic targets plays a different role in the pathogenesis of AD as summarized in Table 2. Amongst them, AChE is regarded as the vital target involved in AD pathophysiology. The inhibition of the active site of AChE prevents degradation of ACh in synapses, which results in an increased concentration of ACh for cholinergic neurotransmission. The enzyme is also found to induce aggregation of Aβ; the peripheral anionic site of AChE can interact with Aβ to facilitate fibril formation [69,70]. Thus, the inhibition of the peripheral anionic site is considered an alternative strategy to hinder Aβ aggregation. To date, many multi-target design strategies involving AChE have been adopted for the development of multi-target ligands against AD.

Based on these targets, libraries of compounds can undergo biological screening to identify hits that exhibit polypharmacological actions. These hits can be structurally modified to optimize the overall profile as rationally as for the compounds derived from the knowledge-based/medicinal chemistry-based approaches. In tandem with the knowledge of pharmacophores with respective biological activities, understanding of the role of different targets in AD progression will certainly facilitate the design of multi-target ligands against relevant targets of interest. 

On top of the knowledge-based and biological screening-based approaches, computational methods are also employed to guide the design of molecular scaffold of potential polypharmacological leads. Pharmacophore modeling, machine learning and structure-based virtual screening are increasingly used to predict biological activity and target–ligand interaction for various libraries of compounds [93,94]. Both pharmacophore modeling and machine learning make use of large bioactivity databases to obtain the likely activity spectra of small molecules based on molecular similarity and patterns. In the structure-based virtual screening, libraries of compounds are computationally screened against targets of known 3D structure to predict the molecular interactions between the target and each chemical compound [93,94]. These computational tools are in fact useful in prioritizing the molecular fragments for rational design of new lead compounds with polypharmacological actions.

In the following section, multi-target ligands for AD discovered through knowledge/medicinal chemistry, biological screening and virtual screening-based approaches are enumerated. 

### 4.1. Medicinal Chemistry-Based Approaches

The medicinal chemistry-based approach makes use of chemical structures of compounds with reported anti-Alzheimer activities as well as that of approved drugs to develop novel multi-targeted ligands for AD. These hybrid compounds are rationally designed by incorporating multiple known pharmacophores (such as those listed in Table 1) into a single drug entity. Together with structural optimization to achieve optimum activity, physicochemical and pharmacokinetic profiles are also taken into consideration in the development of hybrid compounds. Some examples of these compounds categorized as conjugate, merged and fused hybrids are illustrated accordingly in this section. 

#### 4.1.1. Conjugate Hybrids

**Figure 4:**
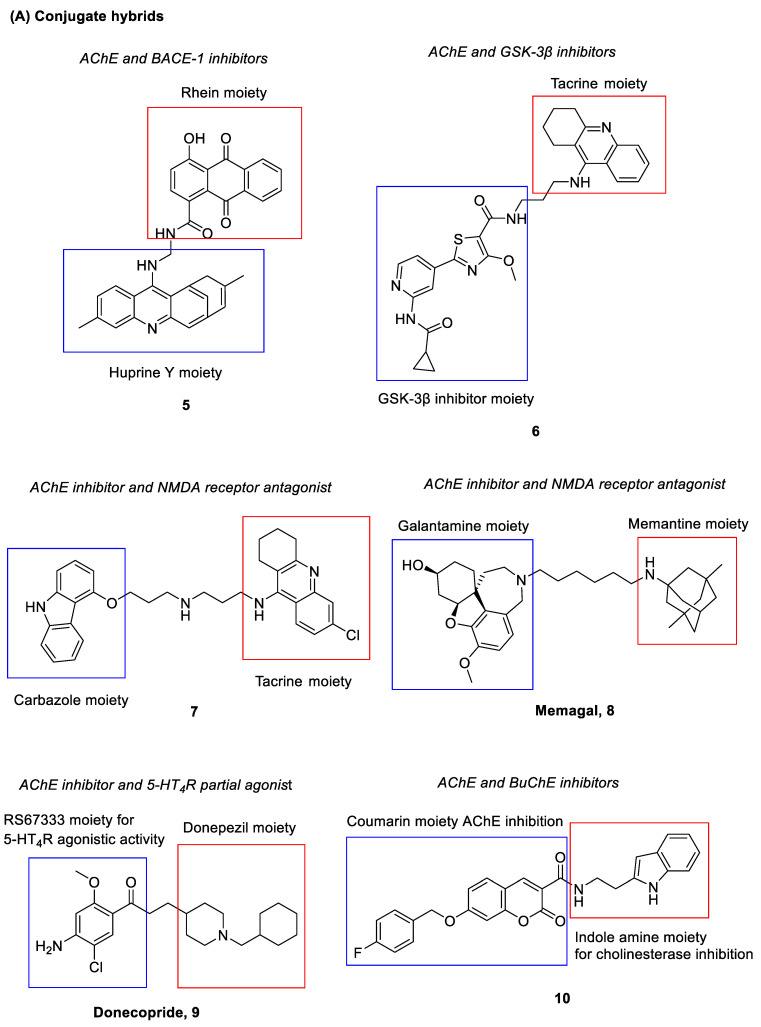

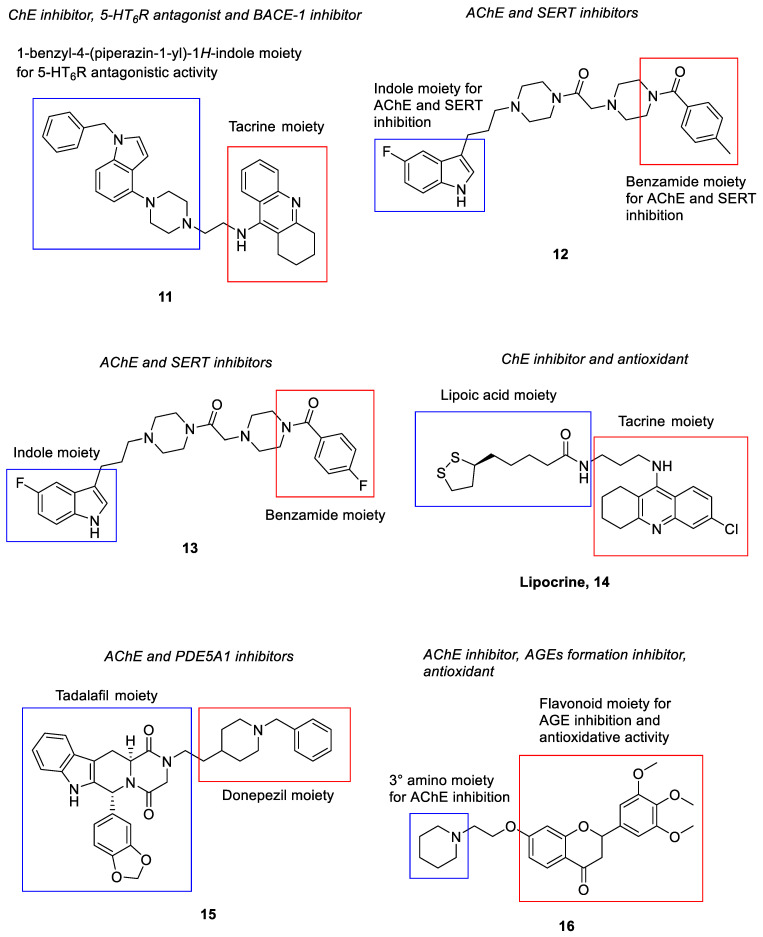

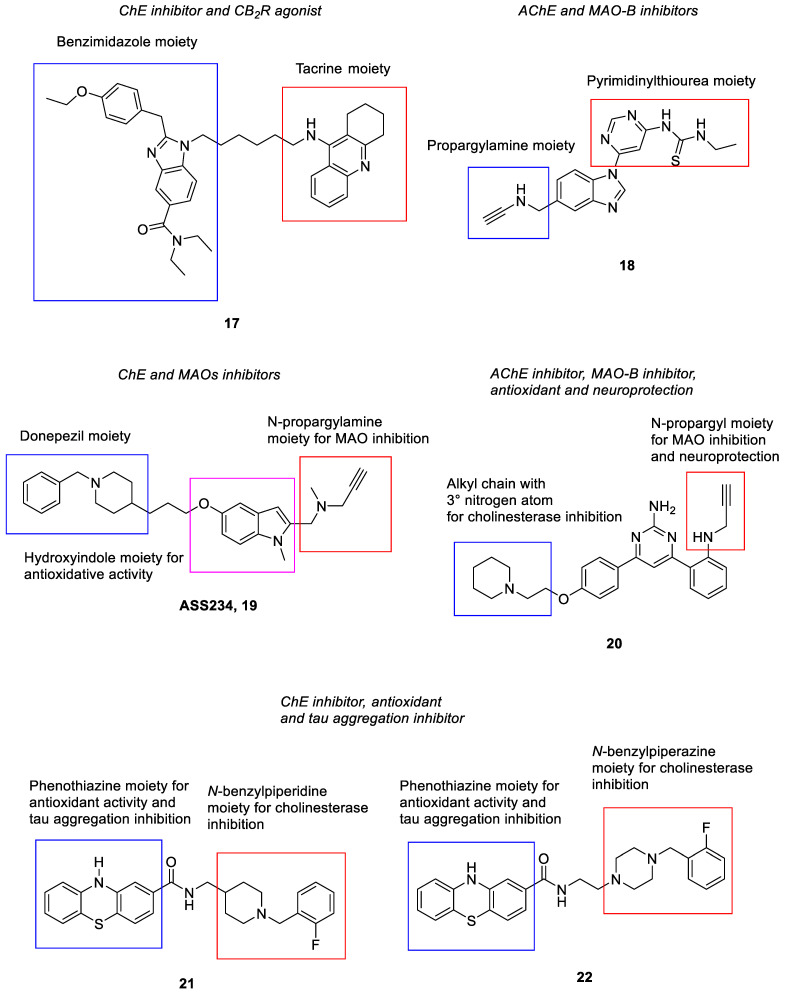

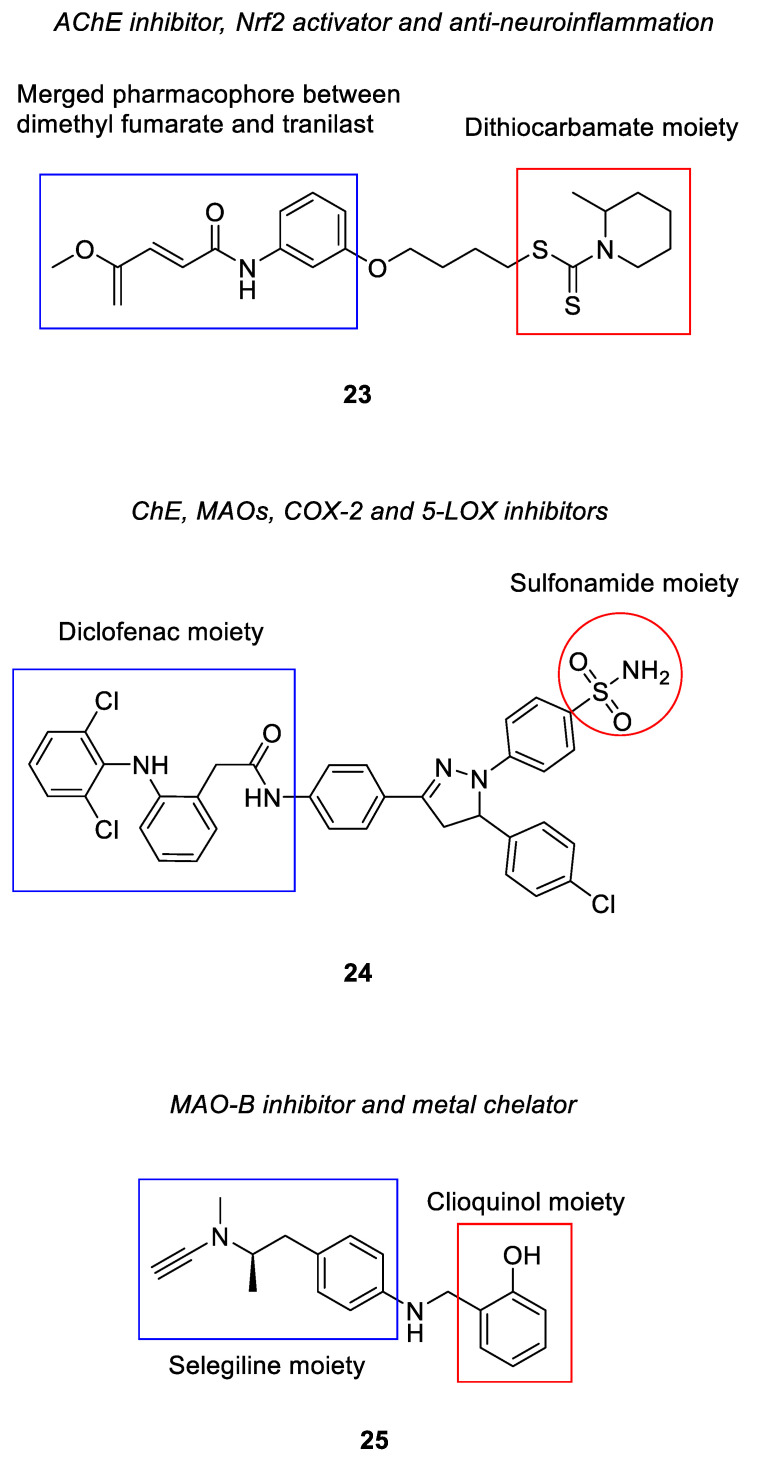

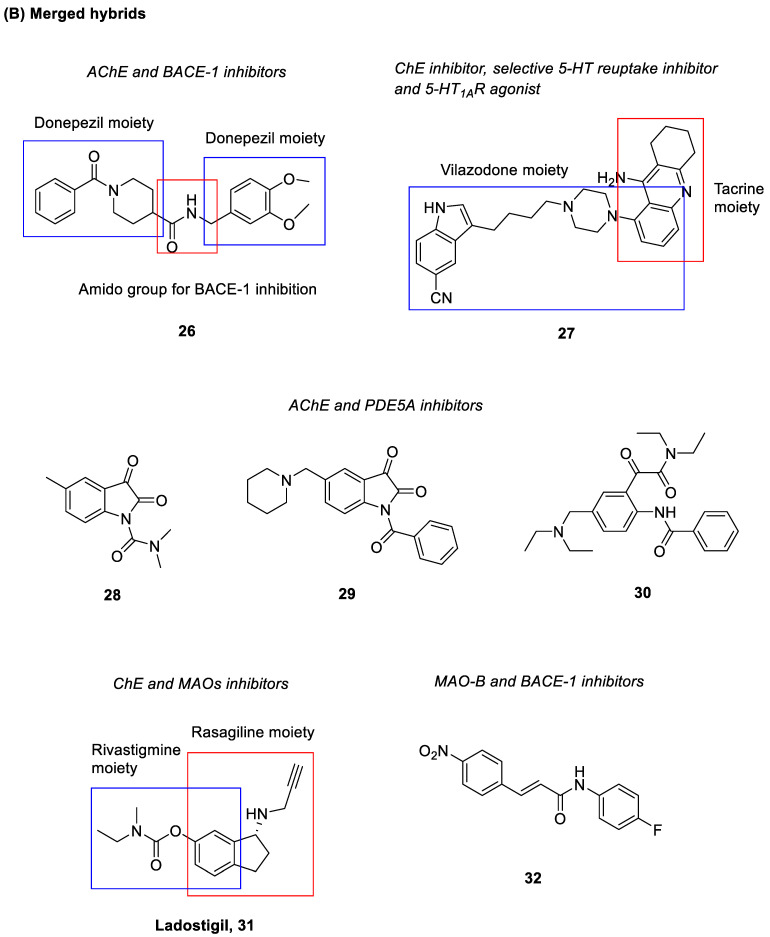

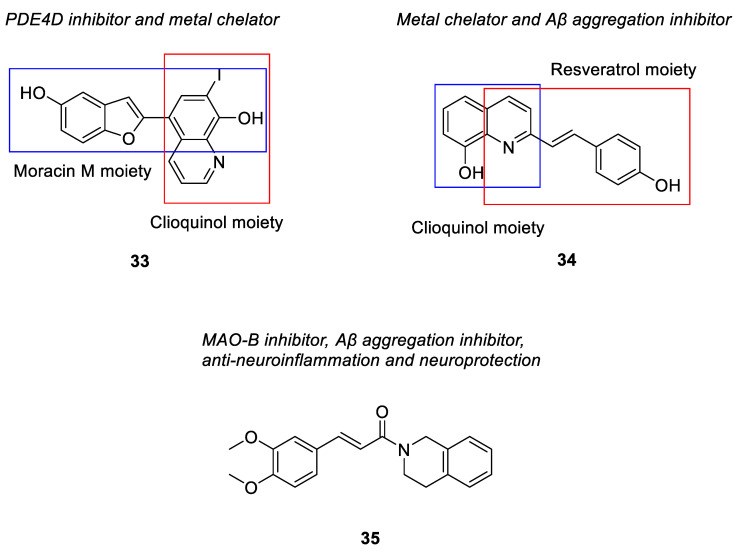

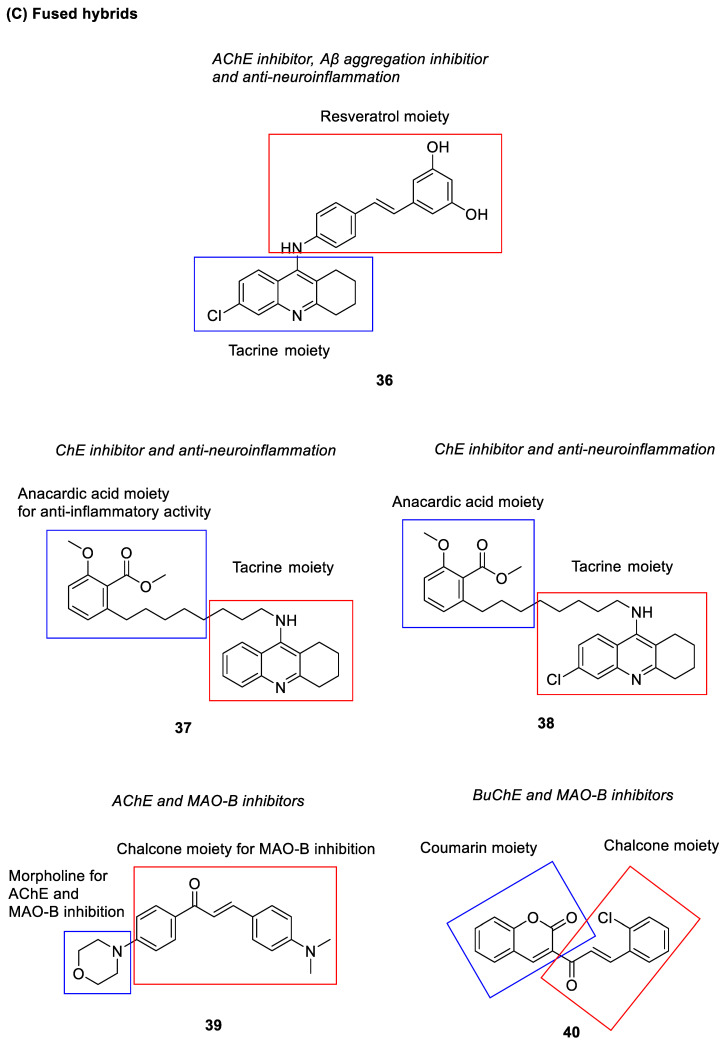
Polypharmacological ligands with anti-Alzheimer activities derived from a medicinal chemistry approach.

Compound **5** (Figure 4) is a polypharmacological ligand that was shown to inhibit both AChE and BACE-1 [95]. Inhibition of AChE blocks the breakdown of the neurotransmitter acetylcholine, while inhibition of BACE-1 decreases the production of the Aβ peptide. Such a compound is derived from an anthraquinone, Rhein and a tacrine-huperzine A hybrid, huprine Y, which are connected by a long alkylamine linker [95]. It demonstrated potent inhibitory activities against AChE (IC_50_ = 3.6 nM) and BACE-1 (IC_50_ = 120 nM). Additionally, it was also found to show 47.9% of Aβ_42_ anti-aggregating activity at 10 µM [95]. 

Compound **6** (Figure 4) was found to exert inhibitory activities against both AChE and GSK-3β. Upon inhibition of GSK-3β, tau hyperphosphorylation is inhibited and it prevents aggregation of tau proteins into neurofibrillary tangles [16]. The compound is made up of a tacrine moiety and another counterpart with GSK-3β inhibitory activity; both moieties are linked by a propyl chain [96]. It showed potent inhibitory activities against AChE and GSK-3β with IC_50_ = 6.5 nM and IC_50_ = 66 nM, respectively, that led to cognitive improvement. When compared to one of its parent compounds, tacrine, compound **7** also showed lower hepatotoxicity [96]. 

Rosini and co-workers had developed a dual acting AChE inhibitor and NMDA receptor antagonist, carbacrine (compound **7**) [97] (Figure 4). It consists of a tacrine moiety and a carbazole ring linked by a dipropylamino chain. The compound showed dual inhibitory activities against AChE and the NMDA receptor with AChE IC_50_ = 2.15 nM and NR1/NR2A IC_50_ = 0.74 μM, respectively. On top of that, it was also found to block in vitro AChE-induced Aβ aggregation and Aβ self-aggregation (36% at 10 μM) as well as to reduce oxidative stress (ROS inhibition, IC_50_ = 23 μM) [97]. 

Memagal (**8**) (Figure 4) is another hybrid compound targeting both AChE and the NMDA receptor. It is derived from galantamine and memantine, which act as AChE inhibitor and NMDA receptor antagonist, respectively [98]; both are linked by a hexyl chain. The compound had demonstrated potent inhibitory activities against AChE with IC_50_ = 1.16 nM. In addition, it also showed an inhibitor constant, K_i_ = 4.6 µM against the NMDA receptor by a [^3^H] MK-801 binding assay [98]. Similar results were obtained for its NR2B-containing NMDA receptor inhibitory activity based on a [^3^H] ifenprodil binding assay with an inhibitor constant, K_i_ = 4.6 µM. Moreover, the compound was shown to possess a potent neuroprotective effect, whereby it inhibited NMDA-mediated neurotoxicity in a SHSY5Y cell viability assay with IC_50_ = 0.28 nM [98]. 

Donecopride (**9**) (Figure 4), a dual acting AChE inhibitor (IC_50_ = 16 nM) and 5-HT_4_ receptor partial agonist (K_i_ = 8.5 nM) was designed and synthesized by Dallemagne and team [99]. It comprises the key pharmacophore of RS67333, a partial 5-HT_4_R agonist and that of donepezil; the two moieties are connected by an ethyl chain. The compound stimulated the non-amyloidogenic 5-HT_4_ receptor-mediated cleavage of APP and promoted neurotrophic sAPP-α release (donecopride, EC_50_ = 11.3 nM versus RS67333, EC_50_ = 27.2 nM). Furthermore, it also possessed favorable bioavailability and druggability profiles. In in vivo studies, it exhibited precognitive effects with an improvement in memory performance observed at 0.3 mg/kg and 1 mg/kg of donecopride administration via intraperitoneal injection [99]. 

As another type of cholinesterase (ChE) enzyme, BuChE shares similar physiological functions as AChE. Studies showed that inhibition of AChE might cause an elevation of BuChE levels in the body, thus suggesting its role in AD pathogenesis. Design and discovery of dual AChE/BuChE inhibitor hybrids has garnered interest from researchers in recent decades [71]. Compound **10** (Figure 4) was discovered from the synthesis and development of N-(2-(1*H*-indol-3-yl)ethyl)-2-oxo-2*H*-chromene-3-carboxamides derivatives, which were tested for their AChE and BuChE inhibitory activities [100]. The scaffold of the compounds consists of a coumarin moiety known for its binding ability to the peripheral anionic site of AChE [101] as well as an indole amine moiety previously reported for its ChE inhibitory activities [102,103]. Out of all derivatives identified, compound **10** showed the best inhibitory activities, with IC_50_ = 0.16 nM for AChE, and IC_50_ = 29.7 nM for BuChE. It was noted that the addition of a benzyloxy moiety at the 7^th^ position of the coumarin moiety could have contributed to an increase in the AChE inhibitory activity. 

A multi-target directed compound (compound **11**) (Figure 4) consisting of pharmacophore fragments of tacrine and 1-benzyl-4-(piperazin-1-yl)-1*H*-indole had been found to demonstrate effective inhibition of ChEs together with serotonergic 5-HT_6_ receptor antagonism (AChE, IC_50_ = 26 nM; BuChE, IC_50_ = 5 nM; 5-HT_6_, K_i_ = 94 nM) [104,105]. It not only displayed well balanced activities against targets of interest but also showed favorable preliminary pharmacokinetic and physicochemical properties [104,105]. The authors have subsequently performed in vitro FRET assays and *in cellulo* studies via an *Escherichia Coli* model of protein aggregation to investigate the β-secretase, tau and Aβ aggregation inhibitory activity of the compound. The results showed that it possessed inhibitory potency of 59% and 56% at 10 μM against tau and Aβ aggregation, respectively, in an *in cellulo* assay as well as IC_50_ = 4 μM against the *h*BACE [106]. This has led to the identification of a potential multi-target ligand with a broad range of biological activities for disease-modifying and symptomatic treatment of AD. 

A new series of indolyl-piperazinyl oxoethyl-benzamido piperazines were developed and evaluated as multi-target ligands against SERT and AChE [107]. The scaffold was designed based on the rationale that both indole and benzamide derivatives had previously been shown to be beneficial for affinity towards the serotonin system [108]. In addition, AChE inhibitory activity was also reported in compounds with similar structural frameworks [109]. Among the new derivatives, compound **12** and **13** (Figure 4) exhibited an AChE inhibition profile (compound **12**, IC_50_ = 3.6 μM; compound **13**, IC_50_ = 3.4 μM) in the same order of magnitude as donepezil (IC_50_ = 2.17 μM) and nanomolar affinity against SERT (compound **12**, IC_50_ = 122 nM; compound **13**, IC_50_ = 212 nM). Of note, the substitution of fluorine at the 5-position of the indole moiety had conferred the SERT nanomolar affinity; such indolic-fluorinated derivatives were also among the best structures for AChE inhibition. Both compounds also demonstrated a low toxicity profile across the range of concentrations studied [107]. 

Lipocrine (**14**) (Figure 4) is a compound with the chemical structure made up of a tacrine analogue and a natural antioxidant, lipoic acid, linked by a propyl chain [110]. The coupling of the two moieties has resulted in a hybrid with a significantly improved biological profile in relation to the parent tacrine and lipoic acid. It could inhibit the activity of AChE and BuChE (AChE, IC_50_ = 0.253 nM; BuChE, IC_50_ = 10.8 nM) and was able to reduce AChE-induced Aβ aggregation. It could also protect neuronal cells against ROS formation; the compound produced strong dose–dependent inhibitory effects towards the formation of ROS with 64% inhibition at 50 μM, and the cell viability was not affected at such a concentration [110].

PDE in the brain is another target that has been found to play a role in cognitive functions. In fact, inhibition of PDE has been found to effectively restore cognitive deficits in AD through regulation of signaling pathways by elevating levels of cAMP and/or cGMP [77]. Hybrid **15** (Figure 4) targeting both AChE and PDE had been synthesized by Mao et al., in which the tadalafil moiety (a selective PDE5 inhibitor) was joined to the 1-benzylpiperidine moiety of donepezil via an ethyl chain [111]. The compound showed selective and considerable inhibitory activities against AChE (IC_50_ = 32 nM) and PDE5A1 (IC_50_ = 1.530 µM) [111]. In vivo studies showed that the citrate of compound **15** reversed cognitive dysfunction in a scopolamine-induced AD mouse model. In addition, it also enhanced cAMP response element-binding protein (CREB) phosphorylation in vivo, leading to improvement in cognitive impairment and restoration of synaptic function in AD. In comparison to the parent compounds, compound **15** showed improved blood–brain barrier (BBB) permeability (permeability coefficient (P_e_) = 9.25 × 10^−6^ cm/s) as well [111]. 

Compound **16** (Figure 4), a flavonoid derivative, was discovered with dual action against AChE and AGEs formation [112]. AGEs are products of non-enzymatic glycosylation of glucose and other non-reducing sugars with protein amino groups. Such end products are perceived to be the cause of chronic complications of diabetes mellitus [113]. Recent studies have found that significant levels of AGEs are immunohistochemically detected in both senile plaque and NFTs from AD brain. On top of hyperphosphorylation of tau proteins, it is suggested that glycation of the tau proteins may facilitate the formation of paired helical filaments; furthermore, the glycation of Aβ peptide has shown to increase self-aggregation as well [84]. The AGEs are also found to provoke the generation of ROS, such as superoxide radicals and hydrogen peroxide [85]. Hence, inhibition of AGEs formation is deemed beneficial in halting the progression of AD. Compound **16** was developed from derivatives of chromen-4-one, in an effort to search for a potential hybrid that can inhibit both AChE and AGEs formation, as well as exerting antioxidant properties [112]. The skeleton of the compound consisted of a flavonoid moiety linked by an ethoxy chain to a piperidine ring; the flavonoid was incorporated to impart the antioxidative and AGEs inhibitory activities, while the tertiary amino moiety (piperidine) was introduced to confer the AChE inhibition. It represented the most promising compound among the derivatives, with an IC_50_ = 5.87 nM for AChE inhibition, an IC_50_ = 23 nM for prevention of AGEs formation, and an IC_50_ = 37.12 nM for radical scavenging activity. Moreover, Compound **16** was also shown to alleviate scopolamine-induced memory deficits in the mouse model [112].

A series of hybrid compounds combining tacrine as ChE inhibitor and benzimidazole as a human CB_2_R agonist via different spacer lengths and structures were designed and synthesized by Scheiner and co-workers [114]. The hybrids showed higher ChE inhibition as compared to tacrine. Radioligand binding studies on human CB_2_R also demonstrated good affinity ranging from nanomolar to less than 10 micromolar for the hybrids. The linker between the two moieties had been associated with a loss of affinity at the hCB_2_R compared to the parent benzimidazole moiety; nevertheless, the hybrids still retained moderate affinity and good selectivity at the human CB_2_R. Among the hybrids, compound **17** (Figure 4) displayed good ChE inhibition and affinity towards human CB_2_R (BuChE, pIC_50_ = 8.7; AChE, pIC_50_ = 7.1; hCB_2_R, K_i_ = 4.5 μM). A cAMP-regulated gene expression assay was carried out and confirmed that the compound maintained the agonist behavior at the human CB_2_R. It also showed considerable inhibition of self- and AChE-induced Aβ aggregation. In the central nervous system, human CB_2_R is indeed abundantly expressed in astrocytes and microglia [79]. Studies revealed that human CB_2_R agonists can suppress the microglia activation and thus the production of neurotoxic factors [80]. As such, microglial activity of the hybrids was also investigated, and compound **17** was found to exhibit an immunomodulatory effect. In in vivo studies, it showed pronounced neuroprotection in the AD mice model at low dosage (0.1 mg/kg, i.p.), and was non-hepatotoxic even at a high dose (3 mg/kg, i.p.) [114]. 

Compound **18** (Figure 4), a dual acting AChE and MAO inhibitor, was discovered from a series of propargylamine-modified pyrimidinylthiourea derivatives [115]. The inhibition of MAO-B could not only lead to anti-oxidative and neuroprotective effects, but could also improve cognitive performance [72,73]. The propargylamine moiety was incorporated to account for the MAO inhibitory activities, while the pyrimidinylthiourea pharmacophore contributed to the AChE inhibition. The compound showed selective inhibitory activities against AChE and MAO-B with IC_50_ = 324 nM and IC_50_ = 1.427 µM, respectively [115]. Consistently, it was found to exert mild antioxidant ability, good copper chelating properties and effective inhibitory activity against Cu^2+^-induced Aβ_1-42_ aggregation. Besides, it was also able to alleviate scopolamine-induced cognitive impairment in the mouse model [115]. 

Another hybrid compound, ASS234 (**19**) (Figure 4) was constructed based on rational combination of benzylpiperidine (from donepezil) and propargylamine for its AChE/BuChE inhibitory activity as well as MAO inhibitory and neuroprotective effect, respectively [116]. Both moieties were linked to a hydroxyindole scaffold, which could impart antioxidative properties. Such a hybrid was found to inhibit AChE, BuChE and MAO activities (AChE, IC_50_ = 0.81 μM; BuChE, IC_50_ = 1.82 μM; MAO-A, IC_50_ = 5.44 nM; MAO-B, IC_50_ = 177 nM). Additionally, it was also able to inhibit Aβ aggregation and possessed antioxidative and neuroprotective properties [116]. The compound is hence regarded as a potential disease-modifying agent for the treatment of AD. 

A new series of *N*-propargylated diphenylpyrimidines were recently identified as multi-target ligands against acetylcholinesterase and MAO-B [117]. The propargyl substituent was introduced into the diphenylpyrimidine skeleton in view of its reported roles in MAO inhibition and neuroprotection [118,119]. Additionally, an alkyl chain with a tertiary nitrogen atom was incorporated in the skeleton as a potential pharmacophore for the acetylcholine/butyrylcholine esterase inhibition; these include the incorporation of piperidine, morpholine, pyrrolidine and *N*,*N*-dimethyl moiety at the *para* position on one of the phenyl rings. Indeed, most of these synthesized compounds were found to exhibit both AChE and MAO-B inhibitory activities with IC_50_ values ranging from sub-micromolar to nanomolar [117]. Compound **20** (Figure 4) showed the most potent AChE inhibitory activity with an IC_50_ value of 0.04 µM and a selectivity index of 626 over BuChE. It also showed good MAO-B inhibitory activity with an IC_50_ value of 0.37 µM. In the ROS production inhibition studies, the compound reduced intracellular ROS levels in SH-SY5Y cells by 32% at 25 µM. It also displayed good neuroprotective potential against 6-hydroxydopamine-induced neuronal damage in SH-SY5Y cells by recovering the cells by up to 77.15% at 25 µM. It was found to be non-toxic against SH-SY5Y neuronal cells at concentrations up to 25 µM. In the in vivo studies, the hydrochloride salt of compound **20** could remarkably attenuate the spatial memory impairment and improve the cognitive deficits in mice. Notably, the salt was able to cross the BBB and reach the target site in the brain tissue upon oral administration as determined from the in vivo BBB permeability test [117].

Phenothiazine-donepezil hybrids were designed and synthesized by Carocci et al. as multifunctional ligands [120]. In such new series of compounds, both *N*-benzylpiperidine and *N*-benzylpiperazine as donepezil-like moieties with different substitutions on the aromatic ring were linked via the one- or two-methylene-amido chains to the phenothiazine moiety. The phenothiazine nucleus is known for its antioxidant properties and inhibitory activities against tau protein aggregation in neurons [121,122]. The hybrids were shown to exhibit antioxidant activities and inhibitory activities against ChEs (AChE and BuChE), in vitro Aβ_1-40_ aggregation and FAAH [120]. FAAH is an enzyme that is involved in the degradation of the endocannabinoid mediator anandamide; in the central nervous system, the endocannabinoid system (ECS) plays an important role in learning and memory, of which its dysregulation is postulated to be one of the possible causes of AD pathogenesis. A study has found that expression of FAAH was elevated during inflammation and neurodegenerative processes [80]. Hence, inhibition of such an enzyme is regarded as another treatment strategy for AD. Among the derivatives, compound **21** and **22** (Figure 4) showed the most promising multi-target activities [120]. Explicitly, compound **21** displayed inhibitory activities against ChEs and FAAH in the range of 1.20 to 5.10 µM. In the dichlorodihydrofluorescein diacetate (DCFH-DA) cell-based antioxidant assay, it showed IC_50_ values of 1.82 and 1.43 µM against HepG2 and SHSY-5Y cells, respectively; in the free radical scavenging activity assay, it demonstrated an EC_50_ value of 0.126 µM. It also moderately inhibited Aβ aggregation with 43% inhibition at a concentration of 10 µM. On the other hand, compound **22** is a more selective AChE inhibitor (AChE, IC_50_ = 0.599 µM; BuChE, IC_50_ = 4.33 µM) and the most potent Aβ aggregation inhibitor (I% = 60% at 10 µM) among all derivatives. In the DCFH-DA antioxidant assay, it showed moderate IC_50_ values of 14.6 and 11.7 µM against HepG2 and SHSY-5Y cells, respectively, while in the free radical scavenging activity assay, it demonstrated comparable scavenging activity as that of compound **21** with an EC_50_ value of 0.231 µM. Both compounds were found to show no cytotoxicity on both HepG2 and SHSY-5Y cell lines at 100 µM upon 1-h exposure [120].

Multi-target ligands based on the pharmacophore structural units of dimethyl fumarate, tranilast and dithiocarbate were recently reported by Guo and team [123]. Dimethyl fumarate (DMF) consists of a α,β-unsaturated ketone moiety, which is a structural feature of nuclear factor erythroid 2-related factor 2 (Nrf2) activator; the compound has also been shown to up-regulate the expression of Nrf2 [124,125]. Nrf2 is one of the components in the Keap1-Nrf2-ARE signaling pathway involved in the defense mechanism of cells against oxidative stress. In the event of oxidative stress, Nrf2 is translocated into the nucleus and initiates transcription of antioxidant genes and phase II detoxifying genes. In addition, its activation is shown to inhibit the induction of pro-inflammatory cytokines and enzymes [87,88]. Therefore, the Nrf2 activator is considered beneficial in regulating the cellular antioxidative and anti-inflammatory processes. Tranilast is an analogue of a tryptophan metabolite that has been shown to attenuate inflammatory responses and alleviate cerebral ischemia–reperfusion injury, suggesting its potential as an anti-inflammatory agent [126,127]. As for dithiocarbamate, the team has previously found that such a moiety could interact with the catalytic active site of AChE and inhibit the enzyme [128]. Structural scaffolds of this series of compounds were afforded through initial merging of both DMF and tranilast moieties; the resulting merged pharmacophore was then linked to the dithiocarbamate via a flexible carbon chain [123]. Various linker lengths and positions as well as terminal tertiary amine were also introduced into the scaffolds to probe their effects on the corresponding biological activities. Out of all derivatives, compound **23** (Figure 4) acquired the most potent inhibitory activity against hAChE with an IC_50_ of 0.053 µM; negligible inhibition was found against the hBuChE [123]. Further mechanistic assays revealed that the compound activated the Nrf2 to exert the anti-oxidative and anti-inflammatory effects. Specifically, the protein expression levels of antioxidant enzymes and phase II detoxifying enzymes were found to be increased upon treatment with compound **23**; pre-treatment with the compound had protected the BV-2 cells from H_2_O_2_-induced cell death and inhibited the accumulation of ROS in the cells. It could also attenuate the LPS-induced inflammatory responses by lowering the levels of pro-inflammatory cytokines and suppressing the expression of pro-inflammatory enzymes, such as inducible nitric oxide synthase (iNOs) and COX-2. In the in vivo studies, the compound was well tolerated at doses up to 2500 mg/kg and was found to ameliorate cognitive deficit in the scopolamine-induced mouse model. It was able to cross the BBB with a P_e_ value of 17.95 × 10^−6^ cm/s as determined from the permeability assay [123].

New diclofenac derivatives had been rationally designed by Javed and team to concomitantly target ChE, monoamine oxidase, COX-2 and 5-LOX [129]. COX-2 is an enzyme that converts arachidonic acid to prostaglandins, which are important inflammatory mediators. Its expression is found significantly increased in the brains of AD patients. The inhibition of COX-2 is thus regarded as beneficial to alleviate neuroinflammation [89]. The AD brain is also found with a large amount of lipoxygenase expression that has been associated with increased Aβ production and tau phosphorylation; studies have shown that 5-LOX inhibitors can reduce the amyloid and tau pathology. In addition, the lipoxygenase has also been linked to oxidative stress in AD patients [90]. These findings suggest that 5-LOX could be another potential target for tackling neuroinflammation. In the attempt to obtain new diclofenac derivatives, the scaffold of diclofenac, a non-steroidal anti-inflammatory drug, was modified by incorporating various rigid (e.g., triazole, pyrazoline) and flexible (alkyl) linkers together with hydrophobic moieties, such as phenyl ring, bicyclic and tricyclic ring at one or both ends of the scaffold, which were found to be favorable towards interaction with active sites of MAOs and AChE/BuChE [129]. On top of that, pyrrolidine, pyrimidine and sulfonamide structural units were also introduced into the scaffold due to their reported multi-target activities related to AD [130]. From the new series of compounds, pyrazoline-sulfonamide derivative **24** (Figure 4) displayed the best multi-target activities with IC_50_ values of 0.03 µM, 0.91 µM, 0.61 µM, 0.01 µM, 0.60 µM and 0.98 µM towards AChE, BuChE, MAO-A, MAO-B, COX-2 and 5-LOX, respectively. It was also found to be non-neurotoxic towards neuroblastoma SH-SY5Y cells in vitro, while in an in vivo acute toxicity study, it was shown safe up to a 2000 mg/kg dose. Through the parallel artificial membrane permeation assay (PAMPA), the compound was found to be BBB penetrant with a P_e_ value of 7.55 × 10^−6^ cm/s [129].

Compound **25** (Figure 4) is structurally derived from the pharmacophore of selegiline and clioquinol, which acts as MAO inhibitor and metal-chelating agent, respectively [131]. The selegiline moiety was conjugated with that of clioquinol via a methylamino chain. The compound showed potent inhibitory activities against MAO-B with IC_50_ = 0.21 µM as well as antioxidant activity with oxygen radical absorbance capacity-fluorescein (ORAC-FL) value of 4.20. Furthermore, it also possessed good metal chelating properties with Cu^2+^, Fe^2+^ and Zn^2+^ ions, suggesting its potential as an antioxidant to scavenge excess metal ions; in turn, it could effectively inhibit Cu(II)-induced Aβ_1-42_ aggregation. The compound was also found to demonstrate good BBB permeability with P_e_ value of 11.5 × 10^−6^ cm/s [131].

#### 4.1.2. Merged Hybrids

A series of donepezil analogues with incorporation of backbone amide group had been revealed to possess dual actions of AChE and BACE-1 inhibitor; the backbone amide groups were introduced into the donepezil scaffold to enhance the BACE-1 inhibition via hydrogen bonding interactions with the catalytic aspartate residues [132]. In particular, compound **26** (Figure 4) displayed potent AChE and BACE-1 inhibitory activities (AChE, IC_50_ = 4.11 nM; BACE-1, IC_50_ = 18.3 nM). Moreover, it showed potential metal chelating properties towards Cu^2+^ and low toxicity on SH-SY5Y neuroblastoma cells. It was also able to cross the BBB as evaluated by a PAMPA study with P_e_ value of 20.13 × 10^−6^ cm/s [132].

Modulation of the serotonergic system has been increasingly regarded as a promising strategy for AD prevention and therapy; particularly, activation of serotonergic neurotransmission was shown beneficial in AD treatment [74,75,76]. Liu and co-workers had discovered a hybrid compound (**27**) (Figure 4) that targeted both ChE and 5-HT receptors [133]. Such a compound consisted of moieties from both tacrine and vilazodone that act as ChE inhibitor and selective serotonin reuptake inhibitor, respectively; the hybrid structure was formed by merging between the phenyl ring of tacrine and piperazine ring of vilazodone. The compound exhibited good inhibitory activities towards 5-HT reuptake, and moderate AChE and BuChE inhibition (5-HT reuptake, IC_50_ = 20.42 nM; AChE, IC_50_ = 1.72 µM; BuChE, IC_50_ = 0.34 µM). Additionally, it also acted as a 5-HT_1A_ agonist with EC_50_ = 0.36 nM. The compound was also shown to be BBB penetrant with P_e_ value of 5.11 × 10^−6^ cm/s [133].

Apart from improving memory performance, inhibition of PDE-5A specifically may also increase cGMP levels, which in turn decrease GSK-3β activity and level of hyperphosphorylated tau proteins in the brain [78]. Zhou et al. had designed, synthesized, and evaluated a new series of indoline-2,3-diones and ring opening derivatives of indoline-2,3-dione as dual AChE/PDE-5A inhibitors [134]. The indoline-2,3-dione (isatin) skeleton was afforded upon modification of scaffolds of both donepezil and Artemisia alkaloid to attain the AChE inhibition; in addition, such a skeleton was postulated to acquire certain inhibitory activities against PDE5A as it shares a similar structure with that of quinazolinone which has been found to exhibit PDE5A inhibition. From the study, they had identified compounds **28–30** (Figure 4) as the most promising compounds, with IC_50_ values of these three compounds ranging between 44.67 nM and 144.50 nM towards the AChE; amongst them, compound **29** showed an IC_50_ of 50 μM against the PDE-5A. These compounds also demonstrated low cell toxicity to A549 cells in vitro [134].

Ladostigil (**31**) (Figure 4) is a compound that was initially developed as an anti-Parkinsonian agent. It was subsequently found to be effective against AD as well. Such a compound was obtained by combining the rasagiline pharmacophore for its MAO inhibition and neuroprotective effects with the carbamate moiety of the ChE inhibitor rivastigmine [135,136]. It exhibited inhibitory activities against AChE, BuChE, MAO-A and MAO-B as well as a neuroprotective effect against oxidative stress (AChE, IC_50_ = 32 μmol/L; BuChE, IC_50_ = 0.48 μmol/L; MAO-A, IC_50_ = 300 μmol/L). Nonetheless, clinical trials had shown that the compound failed in its primary endpoint of halting progression from mild cognitive impairment to AD [137,138].

Venkidath and co-workers had synthesized and evaluated a series of nitro group-bearing enamides for their inhibitory activities against MAOs and BACE-1 [139]. The derivatives consisted of α,β-unsaturated ketone and carboxamide functional groups, which had been shown to be crucial pharmacophores for selective MAO-B inhibition [140]; on top of that, the incorporation of a hydrophobic nitrophenyl group was also found to be favorable towards binding in the MAO-B [141]. Furthermore, the amide group was reported as an important pharmacophore to attain BACE-1 inhibition as demonstrated in some of the BACE-1 inhibitors currently in different stages of clinical trials [142]. Out of these new derivatives, compound **32** (Figure 4) exhibited potent MAO-B inhibitory activity (IC_50_ = 0.0092 µM) and good selectivity towards MAO-B over MAO-A (selectivity index > 1652) [139]. The kinetics studies suggested that it is a reversible and competitive MAO-B inhibitor with a K_i_ value of 0.0049 µM. The compound also showed efficient BACE-1 inhibition with an IC_50_ value of 8.02 µM as compared to the standard inhibitor, quercetin (IC_50_ = 13.40 µM). It could penetrate the BBB with P_e_ = 16.43 × 10^−6^ cm/s based on the PAMPA study [139].

A PDE4D inhibitor, moracin was merged with clioquinol through its benzofuran moiety to afford compound **33** (Figure 4) [143]. The compound not only showed good inhibitory activities against PDE4D (with IC_50_ = 0.32 µM), but also possessed excellent metal chelating ability with Cu^2+^, Fe^2+^ and Zn^2+^ ions. It could also modulate Cu^2+^-induced Aβ aggregation and self-induced Aβ aggregation. Compound **34** (Figure 4), a (*E*)-5-(4-hydroxystyryl) quinoline-8-ol derivative, was obtained by merging the pharmacophore of clioquinol with that of resveratrol [144]. Resveratrol is a naturally occurring polyphenol that has been shown to possess antioxidant and anti-inflammatory properties [145]; it was also demonstrated to be neuroprotective by modulating the activity of microglial cells [146]. The compound was synthesized to endow both metal-chelating property and inhibition of Aβ aggregation. It demonstrated an IC_50_ value of 8.50 μM for inhibition of self-induced Aβ aggregation. Additionally, it could also inhibit copper (II)-induced Aβ aggregation and dissemble the well-structured Aβ fibrils produced from the self- and copper (II)-induced Aβ aggregation [144].

A series of novel *O*-alkyl ferulamide derivatives were designed and synthesized based on the ferulic acid skeleton [147]. The skeleton was shown to display Aβ aggregation inhibitory properties, anti-inflammatory activities, neuroprotective effect, and free radical scavenging activities [148]. Propargyl, benzyl and alkyl fragments known to increase the MAO-B inhibitory activities [149], were incorporated into the skeleton to afford the new *O*-alkyl ferulamide derivatives. They were then evaluated for their MAO-A/MAO-B inhibitory, anti-inflammatory, and anti-Aβ aggregation properties as well as neuroprotective effect. Particularly, compound **35** (Figure 4) demonstrated selective MAO-B inhibitory activities (IC_50_ = 0.73 µM) and good anti-inflammatory activities; it reduced the release of NO and suppressed the TNF-α production in LPS-induced BV-2 cells with an inhibition rate of 62.5% and 55.9%, respectively, upon pre-treatment with 10 µM of compound **35** [147]. It showed considerable inhibition on self-induced Aβ_1-42_ aggregation with 61.7% inhibition of aggregation at 25 µM of compound **35** and Aβ_1-42_. It also exhibited good neuroprotective effect against Aβ_1-42_-induced PC12 cell injury with cell viability increased gradually from 58% to 64% upon pre-treatment with 5 µM and 10 µM of compound **35**, respectively. The compound possessed good in vitro BBB permeation with a P_e_ value of 20.4 × 10^−6^ cm/s and was non-toxic to BV-2 cells at 10 µM [147].

#### 4.1.3. Fused Hybrids

Tacrine-resveratrol fused hybrids were synthesized and evaluated by Jeřábek et al. [150] for their acetylcholinesterase inhibition as well as antioxidant and anti-neuroinflammatory activities derived from tacrine and resveratrol, respectively. Among the hybrids, compound **36** (Figure 4) inhibited the hAChE at micromolar concentration (IC_50_ = 8.8 µM) and effectively blocked the Aβ aggregation with 31.2% inhibition at 50 µM. The presence of the cathecol unit in its structure was deduced to have contributed to the anti-aggregating activity [151]. Besides, the compound also displayed anti-inflammatory and immuno-modulatory activities in neuronal and glial AD cell models; it significantly reduced the nitrite production in a dose–dependent manner from 10 µM to 50 µM and modulated the glial phenotypic pro-inflammatory M1/anti-inflammatory M2 switch. However, the fused hybrids were found to exhibit hepatotoxic effect, which could probably be due to the presence of a tacrine moiety in the scaffold [150].

Hybrids derived from the tacrine analogue and anacardic acid (**37** and **38**) (Figure 4) had been shown to exert ChE inhibitory and anti-neuroinflammatory activities [152]. Presence of a shorter alkyl chain (C8) and methylation of the anacardic acid at both carboxylic and phenolic groups were found beneficial for inhibitory activities against both AChE and BuChE (compound **37**: hAChE IC_50_ = 20.8 nM, hBuChE IC_50_ = 0.0352 nM; compound **38**: hAChE IC_50_ = 2.54 nM, hBuChE IC_50_ = 0.265 nM). Investigation in BV-2 microglial cells revealed anti-neuroinflammatory and neuroprotective activities at low concentration of 0.01 μM for both compounds. BBB permeability of the compounds was estimated through the PAMPA-BBB model; they were found to have the potential to cross the BBB with P_e_ values (compound **37**, P_e_ = 6.99 × 10^−6^ cm/s; compound **38**, P_e_ = 17.70 × 10^−6^ cm/s) matching those of two standard drugs (tacrine, P_e_ = 5.96 × 10^−6^ cm/s; donepezil, P_e_ = 21.93 × 10^−6^ cm/s) known for effective BBB penetration [152].

Morpholine-based chalcone derivatives were recently identified as dual-acting MAO-B and AChE inhibitors [153]. Chalcones have previously been demonstrated to possess good, reversible and selective MAO-B inhibitory activities [154]. Studies reported that the presence of various alkylamino groups on the chalcone A ring had provided AChE inhibitory activity [155]. Moreover, a recent study had shown that the presence of a morpholine ring on chalcone A ring promoted the MAO-B inhibitory activity [156]. Coherently, compound **39** (Figure 4) was found to reversibly inhibit AChE competitively (K_i_ = 2.52 μM) as well as the MAO-B (IC_50_ = 1.31 μM) [153]. The incorporation of a lipophilic group at the *para* position of chalcone ring B, such as -N(CH_3_)_2_ increased the AChE inhibition. The morpholine-based chalcone derivative was also able to cross the BBB as determined through the PAMPA study (P_e_ = 14.44 × 10^−6^ cm/s) and was non-toxic to normal VERO cells [153].

Another series of halogenated coumarin-chalcones has also been reported as multifunctional MAO-B and BuChE inhibitors [157]. Coumarin, a bicyclic compound consisting of an aromatic ring fused with a 6-membered lactone ring, has shown various pharmacological activities, including MAO-B and ChE inhibitory activities [158,159]. Furthermore, the presence and position of electron donating and electron withdrawing groups on ring A and B of chalcone are also found to affect the MAO-B and ChE inhibitory activities [160,161]. Of the derivatives, compound **40** (Figure 4) showed the highest inhibitory activity and selectivity against MAO-B and BuChE (MAO-B, IC_50_ = 0.51 µM, selectivity index over MAO-A = > 78.4; BuChE, IC_50_ = 7.00 µM, selectivity index over AChE = > 5.73) [157]. The nature and orientation of the halogen group, especially the chloro group at the *ortho* position of the phenyl B ring were found to be responsible for the MAO-B and ChE inhibitory activities. Based on the kinetics and reversibility studies, the compound was found to be a reversible and competitive inhibitor of both MAO-B (K_i_ = 0.50 µM) and BuChE (K_i_ = 2.84 µM). It was also able to attenuate H_2_O_2_-induced cellular damage through its reactive oxygen species scavenging effect. The in vitro toxicity studies on Vero cell lines via the MTT assay confirmed that it was non-toxic up to 100 µg/mL, which is approximately equivalent to 100 times its effective concentration in the biological studies [157].

### 4.2. Biological Screening-Based Approaches

Through high-throughput screening, large libraries of compounds are pharmacologically screened at a variety of targets pertaining to AD in search of potential multi-target compounds. Some of the compounds are subsequently structurally optimized to attain optimum biological activities and pharmacokinetic profiles. This target-based approach is particularly beneficial in discovering novel chemotypes and hits for the targets of interest.

**Figure 5:**
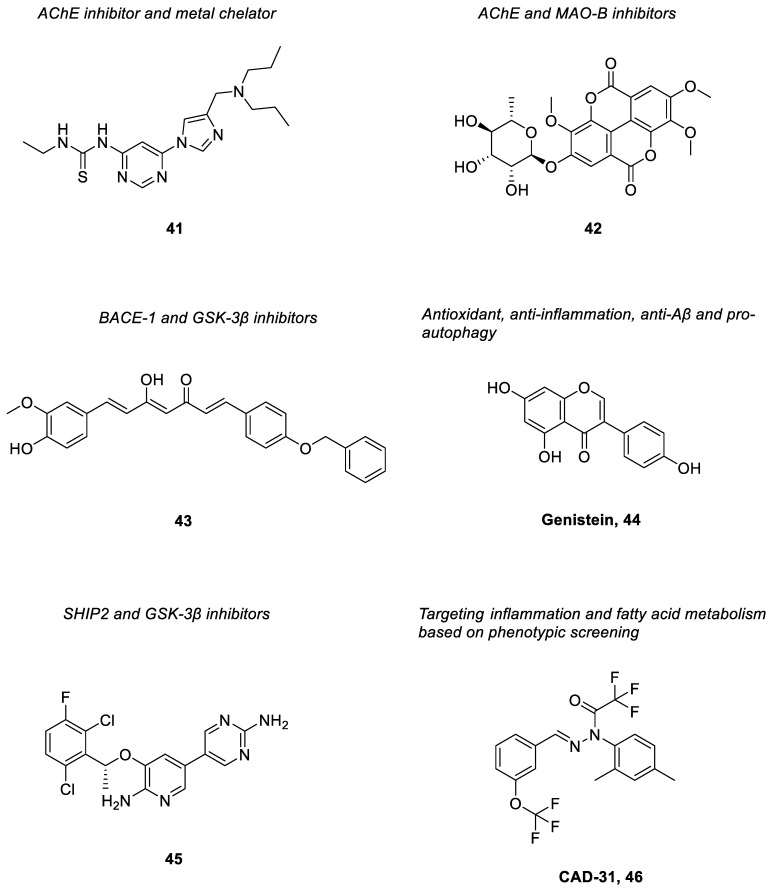
Polypharmacological ligands with anti-Alzheimer activities derived from biological screening approaches.

Upon structural optimization of a hit obtained from a screening approach towards AChE inhibitory activity, a series of non-fused and non-assembly pyrimidinylthiourea derivatives had then been designed and synthesized as novel dual-acting ligands against AChE and metal ions [162]; inhibition of redox-active metals, such as Cu(I/II) that generate cytotoxic reactive oxygen species can help reduce the neuronal damage. Of all the derivatives, compound **41** (Figure 5) exhibited potent inhibition and selectivity against AChE (IC_50_ = 0.204 μM; SI over BuChE > 196) and had a specific metal-chelating ability on Cu^2+^ ions. It showed significant antioxidant effects and modulation of metal-induced Aβ aggregation as well as improved memory and cognitive functions in scopolamine-induced amnesia mice. Moreover, the compound also displayed appropriate BBB permeability both in vitro and in vivo [162].

Oh et al. had isolated ellagic acid derivatives from a methanol extract of *Castanopsis cuspidate* var. *sieboldii* by activity-guided screening [163]. They were evaluated for their inhibitory activities against AChE and MAOs. Of these compounds, 4′-*O*-(α-L-rhamnopyranosyl)-3,3′4-tri-*O*-methylellagic acid (compound **42**) (Figure 5) was found to inhibit AChE (IC_50_ = 10.1 µM) selectively. It also effectively inhibited MAO-B with IC_50_ = 7.27 µM. Based on the enzyme kinetic studies, compound **42** reversibly and competitively inhibited AChE. It showed negligible toxicity towards a normal cell line (Madin–Darby canine kidney (MDCK) cells) at 50 µM [163].

Naturally occurring curcumin derivatives have been screened for a range of activities that can be beneficial in ameliorating AD symptoms. Amongst them, compound **43** (Figure 5) was found to inhibit both BACE-1 and GSK-3β. Both targets have played major roles in the pathogenesis of AD, especially in the production of senile plaques and NFTs [164]. The compound possessed potent inhibitory activities against BACE-1 (with IC_50_ = 0.97 µM) and GSK-3β (with IC_50_ = 0.90 µM). It was able to inhibit Aβ fibril formation and hence prevent Aβ peptide-induced cellular insult. Additionally, it also exhibited neuroprotective activity by inducing the NAD(P)H quinone oxidoreductase 1 (NQO1) enzyme as well as a mild antioxidative effect [164].

Genistein [44], an isoflavone obtained from soybeans, has been found to exert different biological effects beneficial for AD [165]. It was shown to activate the expression of genes coding for antioxidant enzymes, including catalase, superoxide dismutase and glutathione peroxidase in vitro at 0.5 µM [166]; the compound could also reduce oxidation of nucleic acids, lipids and proteins [167]. Oral administration of genistein (10 mg/kg for a week) in LPS-induced animal models has been reported to increase antioxidant activities and reduce lipid peroxidation in the hippocampus [168]. It could inhibit pro-inflammatory cytokine production through regulation of gene transcription of cytokines, such as IL-1β, IL-6, IL-12 and TNF-α [169]; in addition, it inhibited induction of COX-2 transcription and protein expression in cancer cell lines [170] as well as 5-LOX activity in immune cells [171]. Studies also reported that genistein could reduce Aβ levels in the hippocampus and cortex. It was suggested that the compound increased Aβ clearance through activation of APOE synthesis and release [172]; besides, the increased clearance of Aβ peptides could also be due to enhancement of autophagy mediated by genistein with increased LC3-II levels that initiated formation of the autophagosome [173]. The production of Aβ was also found to be reduced through inhibition of β-secretase upon upregulation of protein kinase C (PKC) signaling pathway by genistein [174].

From high throughput screening of a large library of compounds, an FDA-approved drug, crizotinib was identified as a hit compound with SHIP2 inhibitory activity [175]. SHIP2 is a lipid phosphatase that generates phosphatidylinositol 3,4-bisphosphate (PI(3,4)P_2_) from phosphatidylinositol 3,4,5-triphosphate (PI(3,4,5)P_3_), and is involved in many diseases including neurodegenerative diseases [176]. Studies had indicated that inhibition of SHIP2 could reduce hyperphosphorylation of tau protein by the FcγRII receptor and improve memory impairment in mouse models [91,92]. In addition, a relationship between expression of the SHIP2 gene and cognitive decline and Aβ load in AD patients was observed [177]. Hence, SHIP2 is regarded as a potential therapeutic target for AD. Upon extensive structural elaboration of crizotinib, a new series of derivatives had been synthesized [178]. The most potent derivative, compound **45** (Figure 5) showed an IC_50_ = 2.0 μM for the inhibition of SHIP2. Notably, it was also found to inhibit activation of GSK-3β in the HT22 neuronal cells. Such a compound also displayed favorable physicochemical properties, particularly high brain penetration [178].

Phenotypic screening against old age-associated brain pathologies have been employed in AD drug discovery to identify potential hit compounds that showed efficacy in pertinent cell culture assays [179,180]. Following structural modification and optimization of hit compounds obtained from such approaches, compound **46**, CAD-31 (Figure 5) was discovered [181]. It is a novel anti-AD drug candidate that exerted good neuroprotective effect in six distinct nerve cell assays mimicking toxicities observed in the old brain. It was also found to reduce brain inflammation and memory deficit as well as to increase the expression of synaptic proteins in symptomatic AD mice [181]. Consistently, metabolic data from the brain indicated that the major effect of CAD-31 revolves around fatty acid metabolism (energy metabolism) and inflammation, which are the major factors in the pathogenesis of AD [182]. On top of that, it was also shown to be brain-penetrant and safe according to the pharmacological and toxicological studies [181].

### 4.3. Virtual Screening-Based Approaches

Virtual screening is an alternative to high throughput screening that involves costly and time-consuming experimental screening of a library of compounds against targets of interest. Through this computational approach, large libraries of small molecules are screened via in silico methods to identify potential hits, which can be further structurally modified to generate new lead compounds with polypharmacological activities.

**Figure 6:**
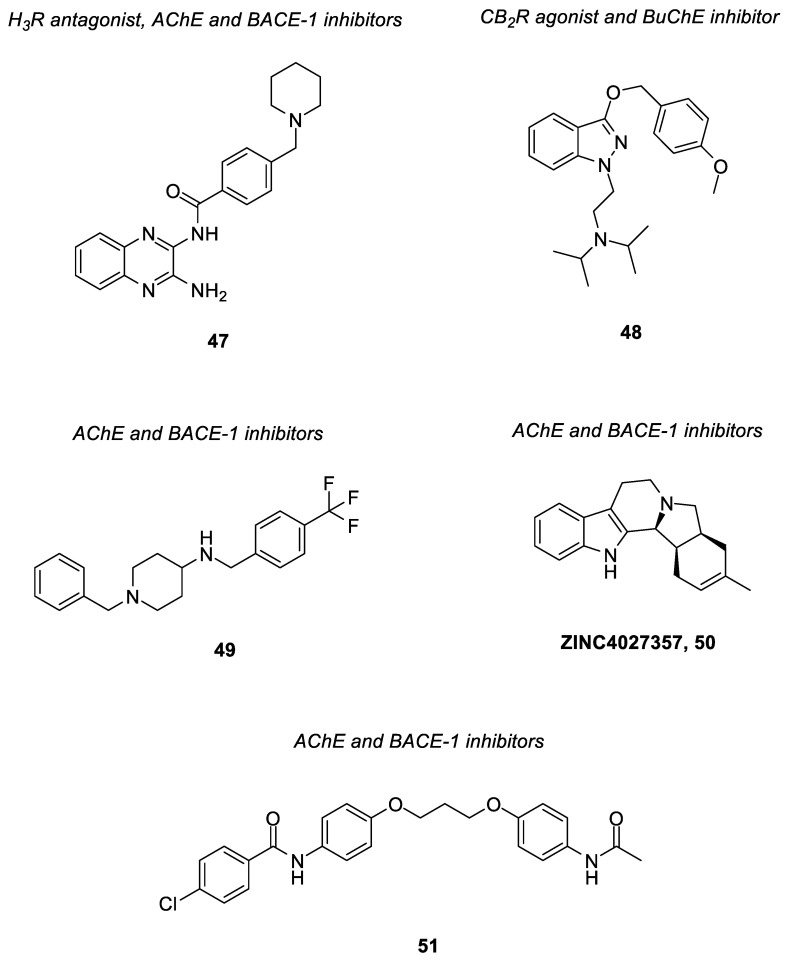
Polypharmacological ligands with anti-Alzheimer activities derived from a virtual screening approach.

Huang et al. had reported a novel series of quinoxaline derivatives as multi-target ligands that inhibited both AChE and BACE-1 as well as antagonized the H_3_ receptor, which was obtained through the virtual screening approaches [183]. Studies showed that activated presynaptic histamine H_3_ receptor decreases the release of ACh from cholinergic neurons [82]; thus, antagonism of H_3_ receptor and inhibition of AChE can ultimately increase synaptic levels of ACh [183]. It was also found that the presence of Aβ increases the AChE activity [83]; simultaneous inhibition of both AChE and BACE-1 will help reduce Aβ generation and hydrolysis of ACh. By taking these into consideration, Huang and co-workers had designed a new scaffold based on 2-amino-3,4-dihydroquinazoline of BACE-1 inhibitor as the core ring moiety of the H_3_ receptor antagonist, and benzyl pyrrolidine fragment of AChE inhibitor, BYYT-25 as the basic center; both moieties were connected by a linker [183]. Subsequently, a virtual database consisting of quinoxaline derivatives was screened on a pharmacophore model of BACE-1 inhibitors built in previous work, and then filtered by a molecular docking model of AChE. Seventeen quinoxaline derivatives were selected, synthesized, and evaluated for their biological activities. Among the derivatives, compound **47** (Figure 6) showed the most potent activity towards H_3_R/AChE/BACE-1 (H_3_R antagonism, IC_50_ = 280 nM; AChE inhibition, IC_50_ = 483 nM; BACE-1 inhibition, 46.64% inhibitory rate at 20 μM) and high selectivity over histamine H_1_, H_2_ and H_4_ receptors [183].

Findings have identified CB_2_R in the brain of AD patient [184], which are mainly expressed in microglial cells. Activation of the CB_2_R can reduce the production of pro-inflammatory molecules by modulating the migration of macrophages [81]; as a result, this may suppress microglia-mediated neurotoxicity. A new series of indazole ether derivatives with dual action as CB_2_ cannabinoid agonists and BuChE inhibitors was designed based on computational methods [185]. In such an approach, a molecular docking study was performed using the CB_2_R model as target against a virtual library consisting of different indazole derivatives. From the modelling results, the derivatives were structurally optimized and subsequently synthesized. The derivatives were then evaluated for the CB_2_ agonistic activity and BuChE inhibitory activity via radioligand binding assays with [^3^H]-CP55940 and in vitro inhibitory assays of BuChE, respectively. Amongst them, compound **48** (Figure 6) had been revealed as a full agonist of CB_2_R with K_i_ = 7.7 μM, and simultaneously showed BuChE inhibition with IC_50_ = 4.8 μM [185]. The compound is therefore regarded as a potential therapeutic agent for the treatment of Alzheimer’s disease.

A structure-based drug design approach was applied by Sharma et al., which made use of e-pharmacophore models of protein structures, namely AChE and BACE-1 that each co-crystallized with N-benzylpiperidine nucleus bearing ligand [186]. These e-pharmacophore models were utilized to screen a library of compounds to identify potential hits. The hits were further filtered through other computational frameworks consisting of virtual screening, docking-post processing and molecular mechanics-generalized born surface area (MM-GBSA) analysis. The identified hit was then used to rationally design a series of new N-benzylpiperidine analogues with improved inhibitory activities against AChE and BACE-1. Among the derivatives, compound **49** (Figure 6) presented significant and balanced inhibition against both targets (hAChE, IC_50_ = 0.11 µM; hBACE-1, IC_50_ = 0.22 µM) [186]. It was shown to inhibit self- and AChE-induced Aβ aggregation at 26.1–50.1% and 61.1–89.0%, respectively. It also ameliorated the scopolamine-induced cognitive impairment in elevated plus and Y-maze rat models. Besides, improvement in Aβ_1-42_-induced cognitive impairment was also observed for the compound in the Morris water maze experiment. Furthermore, the compound possessed high brain permeability (P_e_ = 6.0902 × 10^−6^ cm/s) in the PAMPA-BBB study as well as significant oral absorption based on pharmacokinetic studies. It did not exhibit any neurotoxicity towards SH-SY5Y human neuroblastoma cell lines up to maximum tested concentration of 80 µM [186].

Ivanova and co-workers also had recently employed a combination of computational methods to discover potential dual-acting AChE and BACE-1 inhibitors [187]. Molecular docking and dynamics, artificial neural network and multilinear regression models were performed on the publicly available library from ZINC database. The best predicted candidates were then evaluated experimentally for their AChE and BACE-1 inhibitory activities. Through the assays, compound ZINC4027357 (**50**) (Figure 6) demonstrated inhibitory activity against AChE and BACE-1 at 0.55 μM and 5.2 μM, respectively. Such compounds can be further optimized and developed as potent dual AChE and BACE-1 inhibitors [187].

Virtual screening of the Drug Central Database containing 4199 FDA-approved drugs and drugs approved outside the USA was conducted by Sushant and co-workers via molecular docking on AChE and BACE-1 [188]. Upon docking post-processing, three hits were identified to target both enzymes, namely denopamine, guanethidine and propamidine. Subsequent MM/GSA and a molecular dynamic simulation was performed on these hits; propamidine, an antibacterial and antifungal drug has demonstrated the most promising results with minimum binding free energies and stable binding against both enzymes. Based on such a compound, a series of structurally modified propamidine derivatives by replacement of both terminal amidines with substituted benzamide and acetamide moiety were synthesized and evaluated for their inhibitory activities against both targets. Amongst them, compound **51** (Figure 6) showed remarkable in vitro hAChE and hBACE-1 inhibition (hAChE, IC_50_ = 0.832 µM; hBACE-1, IC_50_ = 0.428 µM) [188]. It also showed good self and AChE-induced Aβ aggregation inhibition at 10 µM in thioflavin T assay (self-induced = 30.85%; AChE-induced = 50.64%) in comparison to donepezil (self-induced = 21.24%; AChE-induced = 33.22%) In in vivo studies, the compound exhibited a remarkable improvement in cognitive dysfunction at 10 mg/kg in scopolamine-induced amnesia mouse models [188].

### 4.4. Other Related Targets for Multi-Target Drug Discovery of Alzheimer’s Disease

So far, the target combination of polypharmacological ligands reported for AD mostly involves the AChE, BuChE, MAO-A/B, BACE-1, oxidative stress and metal chelation. Nonetheless, there are some putative targets related to the multifactorial pathogenesis of AD which are much less explored. These unfathomed targets as illustrated in the following section, are deemed worthwhile for further investigation on their potential combination with other omnipresent targets and multi-target anti-AD drug discovery.

The innate immune cells, microglia, play various roles in the central nervous system, such as immune response, phagocytosis, maintenance of homeostasis and extracellular signaling; microglial targets have been proposed to be critically involved in the early stages of AD via the aforementioned mechanisms. These include toll-like receptors (TLR2 and TLR4), microglial fractalkine receptors (CX3CL1/CX3CR1), receptor-interacting serine/threonine-protein kinase 1 (RIPK1) and purinergic P2X and P2Y receptors; regulation of these targets is shown to confer favorable effects towards AD [189,190]. On the other hand, sigma-1 receptor (S1R), which is highly expressed in neurons has been found to influence neuronal plasticity and neurotransmitter release, rescue endoplasmic reticulum stress and mitochondrial dysfunction as well as impart neuroprotective effects upon its activation [191,192]. It has been proposed that the S1R could be potentially combined with glutamate NMDA and muscarinic receptors for the development of multi-target ligands for AD [193]. Dysregulation of transcription also plays a role in the progression of AD that eventually affects neuronal plasticity, memory and learning. Such dysregulation has been associated with histone acetylation homeostasis that is greatly impaired in neurodegenerative diseases, which shifts towards a state of hypoacetylation. Histone deacetylase (HDAC) is an enzyme involved in deacetylation of histone proteins that promotes chromatin compaction, leading to transcription repression [194,195]. Thus, modulation of HDAC can potentially regulate the process of transcription and improve memory and cognitive deficits as observed in AD.

Enzyme soluble epoxide hydrolase (sEH) metabolizes epoxyeicosatrienoic acids (EETs), the metabolites of arachidonic acid that help to reduce inflammation and oxidative stress. Upon metabolism of EETs, such effects are attenuated. In fact, inhibition of sEH has been shown to exert anti-inflammatory effects and improve cognitive deficits in different AD mouse models; furthermore, its expression is also found upregulated in the brains of AD patients [193,196,197]. Modulation of the enzyme can therefore lead to alleviation of neuroinflammation in AD. Peroxisome proliferator-activated receptor-gamma (PPARγ), a prototypical ligand-activated nuclear receptor, is another target found to be involved in inflammation, oxidative stress and eventual neuronal death. Increased levels of such nuclear receptors are noticed in the brains of individuals with AD. PPARγ ligands have been shown to attenuate degenerative processes in the brain via control of anti-inflammatory mechanisms and oxidative stress [198,199]. It is suggested that the targets associated with the inflammation mechanisms, such as COX-2, 5-LOX, sEH and PPARγ could be combined for their synergistic effects and be considered for the drug design and discovery of multi-target ligands against neuroinflammation.

Kinases are also found to be related to the pathogenesis of AD, of which their functions are interlinked with different signaling pathways. Dysregulation of some kinases, for instance, GSK-3β and Rho-associated coiled-coil kinases (ROCKs) contributes to tau pathology via their regulation of proteins involved in cytoskeleton processes and destabilization of microtubules with resulting synaptic dysfunction in AD [193,200,201]. Another kinase, p38 mitogen-activated protein kinase α (p38α) expressed in neurons is also found to be involved in tau biology and neuronal plasticity; in the microglia and astrocytes, such a kinase regulates brain inflammation, and its inhibition has shown to promote autophagy by microglia [202,203,204]. Modulation of these kinases is therefore regarded as beneficial in tackling the tau hyperphosphorylation in AD.

## 5. Conclusions

In light of the complex network of AD pathological processes, development of multi-target drugs has been considered as an alternative to the currently available single-target drugs and combination therapy. Despite the potential of multi-target drugs in surpassing single target therapy, there are still other aspects which should be taken into consideration in developing multi-target ligands. As AD progresses from primary to secondary stage, and subsequently to the symptomatic stage, combination of therapeutic effects exerted by the multi-target ligands should be in accordance with the given stage of the disease. Target combination for concurrent modulation with multi-target ligands are to be compatible in mechanisms of action; nonetheless, it is also crucial to avoid promiscuous effects arising from interactions with harmful off-target sites. To achieve this, good understanding of pathway-target-drug-disease relationships and adverse events profiling is required. On top of that, it is also crucial to ensure the multi-target ligands possess balanced activity towards targets of interest at the given dose.

Similar to most of central nervous system drugs, AD drug candidates need to fulfil stringent physicochemical properties for BBB penetration by passive diffusion. Generally, multi-target ligands possess higher molecular weights than that of single-target ligands; this results in an increase in size and lipophilicity, which may affect oral absorption of the ligands. Hence, it is not only important for the multi-target ligands to have balanced activities against targets of interest, but also the pharmacokinetic properties shall be optimized for the oral route of administration and permeation across BBB. Use of effective drug delivery systems is another possible alternative to be adopted to enable a drug compound to cross the BBB and accumulate in the brain to exert its drug action. In addition to the in vitro activities and pharmacokinetic profiles, appropriate in vivo activity and toxicity profiles should also be exhibited by the multi-target ligands.

Indeed, different strategies have thus far been employed and are underway to facilitate the drug design and discovery of multi-target ligands for AD. Various computational approaches, such as molecular modelling, machine learning and data mining have been widely adopted in the discovery and optimization of novel ligands with enhanced activity against drug targets. For instance, pharmacophore modeling and in silico fragment-based drug design can be useful by identifying structural features or fragments deemed crucial for the activity of interest via computational methods. Similarly, computational tools can also be applied for optimization of physicochemical and pharmacokinetic properties of potential drug compounds. Some of these computational approaches have given rise to network-based organization of drugs and drug-like compounds databases, which not only enable predictions of drugs off-target leading to repositioning of existing drugs for AD treatment, but also provide insights on possible drug–drug interactions. As an example, rasagiline, a drug for Parkinson’s disease is currently in Phase II clinical trials for treating AD patients with mild cognitive impairment. The emergence of a phenotypic screening method has increasingly gained interest in AD drug discovery based on its potential to address the incompletely understood complexity of diseases. Together with incorporation of disease-relevant mechanisms of action into the screening system, this approach is regarded as an alternative to the omnipresent target-based method and may further expand the biological space available for AD drug discovery. Big data analytics in genomics and proteomics is another area of research that can be applied to identify other biological pathways and new targets involved in the pathogenesis of AD.

As approaches of multi-target ligands open new doors of opportunity in the treatment of AD, they also pose challenges in obtaining new ligands with suitable target combinations, balanced in vitro and in vivo activities as well as optimum pharmacokinetic and toxicity profiles. Nevertheless, in tandem with growing research on mechanism of disease pathogenesis and advancement in multi-target drug discovery of AD, the multi-target ligands clearly hold great promise as potential pharmacotherapy for the management of such a multi-factorial neurogenerative disorder.

## Figures and Tables

**Table 1:** Examples of compounds, their corresponding biological activities and chemical moieties as scaffold for the design of polypharmacological lead compounds for AD.

Compound	Biological Activities	Chemical Moiety
Flavonoid	Antioxidant Anti-inflammatory Inhibition of AChE Anti-aggregation Inhibition of monoamine oxidase (MAO) Metal chelating agent	Polyphenol with chroman-4-one or chromone core system 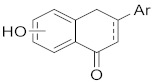
Coumarin	Inhibition of AChE Inhibition of MAO	2*H*-chromen-2-one heterocycle 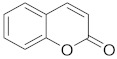
Tacrine	Inhibition of AChE Inhibition of BuChE	9-amino-1,2,3,4-tetrahydroacridine (THA) 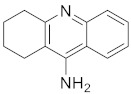
Donepezil	Inhibition of AChE Reduction of neural toxicity of β-amyloid peptide Affinity for nicotinic receptor Neuroprotective action against oxidative stress	Indanone and N-benzylpiperidine 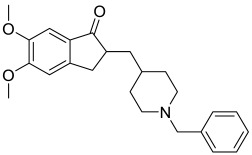
Clioquinol	Metal-chelating agent (Metal-protein-attenuating compound (MPAC)) Disaggregation of β-amyloid peptide; promote its solubilization and clearance	5-chloro-7-iodoquinoline-8-ol 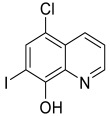
Rasagiline and Selegiline	Irreversible MAO inhibitor with selective inhibition against MAO-B	Propargylamine 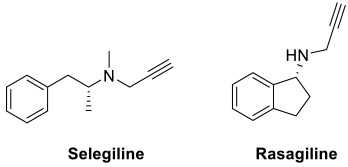
Serotonin and Dopamine	Stimulation of serotonergic receptor (5-HT_1A_ or 5-HT_4_) Activation of α-secretase; promotion of non-amyloidogenic cleavage of APP	Indolamine and phenethylamine fragments 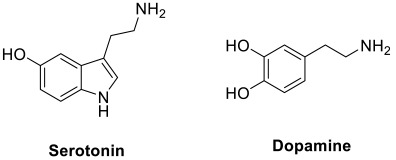
Lipoic acid	Antioxidant with high capacity to scavenge free radicals Increase in acetylcholine level Metal chelating agent Anti-inflammatory	Alpha-lipoic acid (ALA) 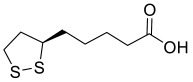
Resveratrol	Anti-inflammatory Decrease in matrix metallopeptidase 9 (MMP-9) Antioxidant Metabolic regulation of AMP-activated protein kinase (AMPK), sirtuin 1 (SIRT1), peroxisome proliferator-activated receptor gamma coactivator-1-alpha (PGC-1α)	Polyphenolic phytoalexin 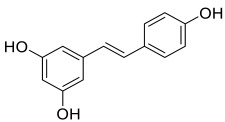
Ferulic acid and caffeic acid	Antioxidant Anti-inflammatory	3,4-dihydroxycinnamic acid 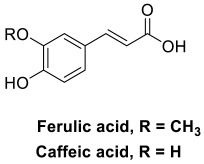

**Table 2:** Therapeutic targets of AD and their role in AD pathogenesis.

Therapeutic Target	Role in AD Pathogenesis	References
AChE	To hydrolyze ACh in synapses To form complexes with Aβ peptide, modify its conformation and promote its aggregation to form β-amyloid plaques	[25,28]
BuChE	To hydrolyze ACh and the concentration of BuChE is found to be increased in advanced AD	[71]
BACE-1	To work with γ-secretase to degrade APP and generate Aβ peptide	[15]
GSK-3β	To hyperphosphorylate Tau proteins, separate them from microtubules and aggregate them into insoluble NFTs To regulate γ-secretase to induce Aβ peptide formation	[16,19]
MAO: MAO-A and MAO-B	To catalyze oxidative deamination To increase production of hydrogen peroxide and ROS To cause oxidative injuries and a toxic environment of neurodegeneration	[72,73]
Metal ions	Excess metals in brain cause peptide aggregation and oxidative stress Dysregulation of Cu^2+^ and Zn^2+^ induces generation of toxic Aβ oligomers by binding to Aβ peptides	
NMDA receptor	To modify major forms of synaptic plasticity that contribute to learning and memory, and to consolidate short-term memory into long term memory Its overstimulation by excess glutamate will cause excitotoxicity and cell death Appropriate inhibition will improve the condition of AD patients	[23,24]
5-HT receptor (serotonergic receptor)	5-HT_1A_ receptor has a therapeutic role in depressive disorder; the agonist and antagonist could be potential therapies for AD 5-HT_4_ receptor partial agonist can enhance ACh release, promote non-amyloidogenic cleavage of APP, form neurotrophic human sAPP-α fragments and decrease Aβ secretion 5-HT_6_ receptor antagonist can alleviate AD symptoms by enhancing cholinergic neurotransmission	[74,75]
SERT	To transport serotonin from the synaptic cleft back to the presynaptic neuron To terminate serotonergic signaling through reuptake of neurotransmitters into presynaptic neuron	[76]
PDE	To hydrolyze and degrade secondary messengers including cAMP and cGMP Regulators of signal transduction in neuroplasticity and neuroprotection Its inhibition can decrease the GSK-3β activity and level of hyperphosphorylated tau proteins	[77,78]
CB_2_ receptor	Activation of CB_2_ receptor reduces production of pro-inflammatory molecules by modulating migration of macrophages	[79,80,81]
H_3_ receptor	Activation of H_3_ receptor (autoreceptor and heteroreceptor) provides negative feedback in the histaminergic system Its activation also inhibits release of other neurotransmitters	[82,83]
AGEs	Glycation of tau proteins, leading to formation of paired helical filaments Glycation of Aβ peptide, resulting in increase in self-aggregation To provoke generation of reactive oxygen species	[84,85]
FAHH	To degrade endocannabinoid mediator, anandamide; endocannabinoid system in CNS plays a crucial role in learning and memory Its expression is elevated during inflammation and neurodegenerative processes	[86]
Nrf2	One of the components in Kelch-like ECH-associated protein 1 (Keap1)- nuclear factor erythroid 2-related factor 2 (Nrf2)-antioxidant response element (ARE) signaling pathway involved in defense mechanisms of cells against oxidative stress To initiate transcription of antioxidant genes and phase II detoxifying genes Its activation inhibits the induction of pro-inflammatory cytokines and enzymes	[87,88]
COX-2	To convert arachidonic acid to prostaglandins, which are the important inflammatory mediators; the level of prostaglandins is found increased in the frontal cortex of AD patients Its expression is remarkably elevated in the brains of AD patients	[89]
5-LOX	Its expression is found elevated in AD brains which has been associated with increased Aβ production and tau phosphorylation Its inhibition can reduce the amyloid and tau pathology as well as to improve cognitive impairment Association with oxidative stress in AD patients	[90]
SHIP2	Its inhibition reduces hyperphosphorylation of tau protein by FcγRII receptor	[91,92]

## Data Availability

Not applicable.
